# Serum miR-195-5p Exhibits Clinical Significance in the Diagnosis of Essential Hypertension with Type 2 Diabetes Mellitus by Targeting DRD1

**DOI:** 10.6061/clinics/2021/e2502

**Published:** 2021-08-23

**Authors:** Yueyan Hu, Qian Li, Leisheng Zhang, Lianmei Zhong, Man Gu, Bo He, Qiu Qu, Yaling Lao, Kunli Gu, Bingrong Zheng, Hongju Yang

**Affiliations:** I Division of Gastroenterology, The First Affiliated Hospital of Kunming Medical University, Kunming, 650032, China.; II Transfusion Medicine Research Department, Yunnan Kunming Blood Center, Kunming, 650500, China.; III School of Medicine, Nankai University, Tianjin, 300071, China.; IV Joint laboratory of Tianjin University and Health-Biotech, Health-Biotech (Tianjin) Stem Cell Research Institute Co., Ltd., Tianjin, 301700, China.; V School of Medicine, Yunnan University, Kunming, 650091, China.

**Keywords:** Essential Hypertension (EH), Type 2 Diabetes Mellitus (T2DM), miR-195-5p, Single Nucleotide Polymorphism (SNP), DRD1

## Abstract

**OBJECTIVES:**

Diagnosis and management of essential hypertension (EH) or type 2 diabetes mellitus (T2DM) by combining comprehensive treatment and classificatory diagnosis have been continuously improved. However, understanding the pathogenesis of EH patients with concomitant T2DM and subsequent treatment remain the major challenges owing to the lack of non-invasive biomarkers and information regarding the underlying mechanisms.

**METHODS:**

Herein, we collected 200 serum samples from EH and/or T2DM patients and healthy donors (N). Gene-expression profiling was conducted to identify candidate microRNAs with clinical significance. Then, a larger cohort of the aforementioned patients and 50 N were used to identify the correlation between the tumor suppressor miR-195-5p and EH and/or T2DM. The dual-luciferase reporter assay was used to explore the target genes of miR-195-5p. The suppressive effects of miR-195-5p on the 3′-UTR of the dopamine receptor D1 (DRD1) transcript in EH patients with concomitant T2DM were verified as well.

**RESULTS:**

Compared with that in other groups, serum miR-195-5p was highly downregulated in EH patients with concomitant T2DM. miR-195-5p overexpression efficiently suppressed DRD1 expression by binding to the two 3′-UTRs. Additionally, two single nucleotide polymorphisms, including 231T-A and 233C-G, in the miR-195-5p binding sites of the DRD1 3′-UTR were further identified. Collectively, we identified the potential clinical significance of *DRD1* regulation by miR-195-5p in EH patients with concomitant T2DM.

**CONCLUSIONS:**

Our data suggested that miR-195-5p circulating in the peripheral blood served as a novel biomarker and therapeutic target for EH and T2DM, which could eventually help address major challenges during the diagnosis and treatment of EH and T2DM.

## INTRODUCTION

Essential hypertension (EH)—defined as the unexplained rise in blood pressure—is a major cause of cardiovascular and cerebrovascular diseases with a worldwide prevalence ranging from 26.4% in 2000 to 29.2% in 2025 that is mainly attributable to genetic and environmental factors (1,2). For decades, longitudinal studies have indicated the involvement and clusters of predisposing factors, such as being overweight, the aging process, hyperlipidemia, and insulin resistance-associated type 2 diabetes mellitus (T2DM), yet the precise pathogenesis of EH requires further understanding (2,3). For instance, multifaceted T2DM and/or complication-associated inducements, such as hyperglycemia, insulin resistance, excess fatty acids, and the malfunction of pancreatic beta cells are sufficient for inducing thrombosis, vasoconstriction, vascular inflammation, and atherogenesis, which collectively result in the development of EH-associated cardiovascular diseases (3-5). Moreover, more than 39% of the patients with newly diagnosed T2DM were more hypertensive than normotensive patients, whereas EH occurred in up to 75% of the adult T2DM patients (6,7). Long-term EH increases the risk of coronary heart disease, stroke, heart failure, and peripheral vascular disease (7).

MicroRNAs (miRNAs) are small, conserved, endogenously-initiated non-coding RNAs and play a crucial role in multifaceted biological processes, including cell proliferation, apoptosis, development, and tumorigenesis (8,9). Briefly, miRNAs play multidimensional roles in mRNA degradation or post-transcriptional inhibition of expression (8,10). Over 5300 human genes are coordinatively modulated by numerous miRNAs and computational predictions indicate that each microRNA may target thousands of genes (11,12). Recently, emerging evidence has indicated the possibility of using serum/plasma miRNAs—including miR-29b, miR-126, miR-146, miR-130b, and miR-320a—as onset or prognostic biomarkers of T2DM or EH (13,14). Generally, miRNAs function by binding to the recognition element in the 3'-UTR of the target genes, thereby triggering the degradation of the mRNA or inhibiting its translation, negatively regulating the expression of the target mRNA and influencing various physiological and pathophysiological processes (15). Furthermore, increasing numbers of miRNA-based therapies are being successfully employed in murine disease models (16). Circulating miRNAs with differential expression in blood are recognized as novel diagnostic markers and potential therapeutic targets for predicting T2DM or EH. miRNA dysfunction in the pathogenesis of EH combined with T2DM remains to be fully elucidated (9,14).

In this study, we used the miRNA qRT-PCR array to evaluate the expression profile of serum miRNAs among Chinese patients with EH and/or T2DM and healthy donors. Bioinformatic analysis, qRT-PCR validation, and promoter activity detection revealed that miR-195-5p exhibited clinical significance in the aforementioned 50 EH patients with T2DM by directly targeting the 3′-UTR of dopamine receptor D1 (DRD1) and inducing its subsequent downregulation. Furthermore, the variation and susceptibility of two major single nucleotide polymorphisms (SNPs) in the miR-195-5p-binding site in the 3′-UTR of DRD1 were identified in Chinese individuals with EH and T2DM.

## MATERIALS AND METHODS

### Subjects and study design

This study was conducted in accordance with principles and guidelines of Declaration of Helsinki, and approved by the Ethics Committee of First Affiliated Hospital of Kunming Medical University, China (approval number: 2020-L-12). All blood samples were collected from participants after obtaining written informed consent. Generally, the participants were divided into four groups, *i.e.*, EH patients (E), type 2 diabetes mellitus patients (D), E with D (ED), and unrelated healthy individuals (negative controls (N)). Fifty individuals per group were recruited at the First Affiliated Hospital of Kunming Medical University from January 2017 to March 2018. Of the participants, five individuals from each group (age and sex difference<12 months) were randomly selected for serum miRNA expression profile analysis. The differentially expressed miRNAs were screened by bioinformatic analysis and validated by qRT-PCR (for all 50 patients). The general information of representative patients (E, D, ED) and healthy donors (N) is listed in Supplementary Information: Supplementary Table S1.

### Inclusion criteria and exclusion criteria

Diagnosis of diabetes mellitus was made in accordance with the recommendations of the American Diabetes Association (ADA), *i.e.*, fasting plasma glucose (FPG)≥7.0 mmol/L; or HbA1c≥6.5%; or oral glucose tolerance test (OGTT) for 2h blood glucose≥11.1mmol /L. Fasting was defined as not eating or drinking (except water) for at least 8h (17).

EH was diagnosed according to the 1999 World Health Organization (WHO) criteria, *i.e.*, diastolic blood pressure (DBP)≥90 mmHg or systolic blood pressure (SBP)≥140 mmHg, and no antihypertensive medication (18).

Exclusion criteria included type 1 diabetes, secondary hypertension, acute myocardial infarction, severe liver and kidney dysfunction, cancer, use of drugs that may affect HRV, development of a non-sinus rhythm (such as atrial flutter or atrial fibrillation) or use of a pacemaker.

### Plasma collection from the peripheral blood

Whole peripheral blood was collected as previously reported (19). Briefly, plasma was isolated from whole peripheral blood using anticoagulant tube, followed by centrifugation at 3000 rpm for 10 min at room temperature (25°C). After phase separation, plasma was collected using a micropipette, divided into two parts, and frozen at -80°C for subsequent analyses.

### Blood sugar status assessment

The fasting blood glucose (FBG), 2h postprandial glucose in OGTT, fasting insulin, and HbA1c were measured. The homeostasis model assessment-insulin resistance index (HOMA-IR) was calculated using the following formula: HOMA-IR=FBG × fasting insulin/22.5.

### Measurement of blood pressure

Blood pressure was evaluated in subjects who had been seated and rested for 5 min by the nurses in the First Affiliated Hospital of Kunming Medical University. The measurement was performed twice and the mean value was calculated.

### Plasma miRNA enrichment

Total plasma RNAs, including miRNAs were extracted from the plasma samples using TRIzol (Invitrogen, Carlsbad, CA, USA), as previously described, with some modifications (19-21). Briefly, the absorbance (A_260/280_>1.70) and concentration of RNA samples were determined using a NanoDrop ND-1000 spectrophotometer (Thermo Fisher Scientific). RNA integrity was evaluated using agarose gel electrophoresis.

### miRNA qRT-PCR array

For miRNA qRT-PCR array, ∼20 ng total RNA was reverse transcribed into cDNA using the microRNA Reverse-Transcription kit and the RT Primer Pools (Exiqon A/S, Vedbaek, Denmark), according to the manufacturer's instructions. Then, miRNA qRT-PCR was conducted on an ABI PRISM7900 system and miRCURY LNA™ Universal RT microRNA PCR system (Applied Biosystems; Thermo Fisher Scientific). The detailed procedure is available in the Supplementary Information and the primers used for miRNA amplification are listed in Supplementary Table S2.

### Validation of miRNA expression by qRT-PCR analysis

cDNA was synthesized using the miScript Reverse Transcriptase Kit (Qiagen, Germany), according to manufacturer's instructions. The expression levels of candidate miRNAs (miR-197-5p, miR-130a-5p, miR-27a-5p, miR-195-5p, U6) were quantified by qRT-PCR; the miScript SYBR^®^-Green PCR kit (Qiagen GmbH) and ABI PRISM7900 system (Applied Biosystems; Thermo Fisher Scientific) were used for the same. The relative miRNA expression level was determined using the 2^−ΔΔCT^ method, with several modification (3,22-24). The detailed procedure is available in the Supplementary Information, primers used for miRNA amplification are listed in Table S2, and the significantly upregulated, downregulated, and total miRNAs are listed in Supplementary Table S3−S4 and [Table t05] Table S6.

### Luciferase reporter assay

The 3′UTR of the DRD1 comprising the putative miR-195-5p binding sequence and corresponding mutant segment were constructed into the pmir-RB-REPORT™ vector (RiboBio Co., Ltd., Guangzhou, China), according to the manufacturer's instructions. Then, 3×10^5^ HEK-293T cells were seeded into 24-well plates in Dulbecco's modified Eagle's medium (DMEM) containing 10% FBS for 24h before transfection. Luciferase constructs, miR-195-5p mimics, or a negative control were co-transfected into HEK-293T cells using Lipofectamine 2000 (Invitrogen; Thermo Fisher Scientific). A dual-luciferase reporter assay was performed 48h after transfection (Promega). The activities of Renilla and Firefly luciferase were determined by the dual-luciferase reporter system (Promega), according to manufacturers’ instructions (21,25). Three independent experiments were performed. The detailed procedure is available in the Supplementary Information.

### Total protein collection and western-blot assay

RIPA peptide lysis buffer (Beyotime Biotechnology, Jiangsu, China) supplemented with 1% protease inhibitors (Pierce) was used for extracting total protein from HEK-293T cells (48h post-transfection), according to the manufacturer's instructions. The proteins were denatured and then separated by electrophoresis on a 12% SDS-PAGE gel. They were transferred onto on a PVDF membrane (Millipore, Ireland). Thereafter, the membranes were incubated with primary antibodies against DRD1 (ab216644, Abcam) or β-actin (4967S, Sigma) for 12h at 4°C, followed by incubation with HRP-labeled mouse IgG secondary antibody (HAF007, R&D Systems) for 1h at 4°C. The ECL Detection Reagent (ThermoFisher) and Super-signal West Pico Chemiluminescent Substrate (Prierce) was utilized to develop the blot (21,25,26). Finally, gray-scale analysis of the detected protein bands was performed using ImageJ (version 1.8.0, Rawak Software, Inc., Germany).

### Genotyping

Genotyping was performed for the miR-195-5p-binding site in the 3′-UTR of DRD1. Peripheral blood samples were collected and subjected to genomic DNA isolation using the standard phenol-chloroform extraction method, with several modification (21). The purity (A_260/280_) and concentration of genomic DNA were determined using a Biophotometer (Eppendorf, Germany). ABI Prism^®^ 7900HT (Applied Biosystems) was used for PCR and sequencing, according to the manufacturer's instructions. The following primers were used to amplify miRNAs: 5′-AGACCCTTGGAGAAGCTGTC-3′ (upstream) and 5′-GGAAATGCAGGGTTTGAG-3′ (downstream). The SNP locus mutations (231T-A, 233C-G) in the miR-195-5p-binding site of DRD1 3′-UTR are listed in Supplementary Table S5.

### Statistical analysis

All statistical analyses were conducted, as previously reported (20,23,25,28). SPSS 21.0 (SPSS Inc.) was used for the statistical analyses. The data are presented as the mean±standard deviation (SD). Differences between two groups were compared using the Student’s *t-*test. One-way analysis of variance (ANOVA) was performed to compare the differences among three or more groups. Enumeration data are expressed as percentages or rates, and comparisons between groups were performed using the chi-square test. *p*<0.05 was considered significant (NS, not significant; *, *p*<0.05; **, *p*<0.01; ***, *p*<0.001).

### Ethics

All procedures performed in the current study were approved by the Ethics Committee of the First Affiliated Hospital of Kunming Medical University (Approval number: 2020-L-12). Hence, the procedures were in accordance with the Helsinki Declaration and the ethical standards established by the committee of the abovementioned institution responsible for human experimentation (institutional). Written informed consent forms were obtained from all participants or their guardians.

## RESULTS

### Minimal parameters exhibited satisfactory correlation in diagnosis of EH with T2DM

**Figure 1:**
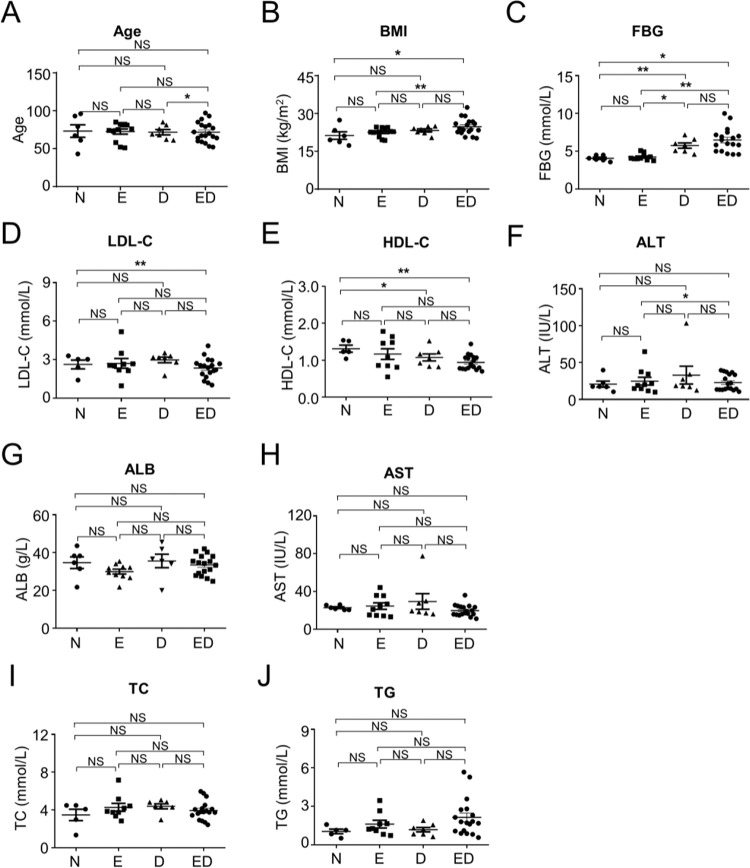
Clinicopathological parameters involved in E, D, ED, and N groups. (**A**) The comparation of age distribution among E (essential hypertension), D (type 2 diabetes mellitus), ED (patients with essential hypertension and concomitant type 2 diabetes mellitus), and N (healthy donors) groups. (**B-J**) Comparations of clinicopathological parameters among N, E, D, and ED groups, including BMI (**B**), FBG (**C**), LDL-C (**D**), HDL-C (**E**), ALT (**F**), ALB (**G**), AST (**H**), TC (**I**), and TG (**J**). All data are shown as Mean±SEM. *, *p*<0.05; **, *p*<0.01; NS, not significant.

**Figure S1:**
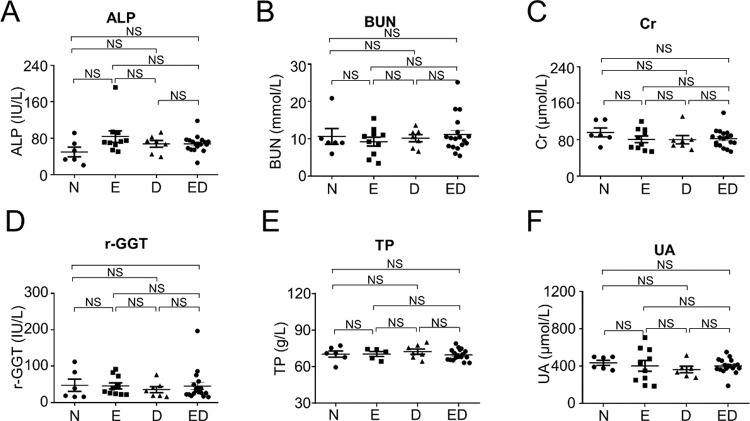
Clinicopathological parameters involved in E, D ED, and healthy donor (N) groups. **A-F** ED-associated clinical parameters in E, D, ED, and N groups, including ALP (**A**), BUN (**B**), Cr (**C**), r-GGT (**D**), TP (**E**), and UA (**F**). All data are shown as the mean±SEM. NS, not significant.

For decades, researchers have been assiduously attempting to optimize the treatment program and evaluating the curative effect for E, D, or ED patients. Consequently, we enrolled 150 patients (50 E, 50 D, 50ED) and 50 unrelated healthy individuals (50 N) in the First Affiliated Hospital of Kunming Medical University during 2017 and 2018. There were no significant differences in age among the groups, except the D and ED patients (Figure 1A). Although multiple parameters, such as body mass index (BMI), fasting blood-glucose (FBG), low density lipoprotein cholesterin (LDL-C), and high density lipoprotein cholesterol (HDL-C) in the N group were different from those in ED patients, differences also occurred among E, D and ED patients (Figures 1B−1F). Furthermore, as shown by the statistical analyses of other clinicopathological parameters involved in clinical trials for comprehensive diagnosis, the current differentiation in the similarities and differences among the aforementioned patients were far from satisfactory (Figures 1G−1J, Figures S1A-S1F, Supplementary Table S1). Hence, there is an urgency for the exploration of more convenient, precise, and non-invasive means for distinguishing ED patients from other groups.

### Characterization of serum miRNA expression profiling

**Figure 2:**
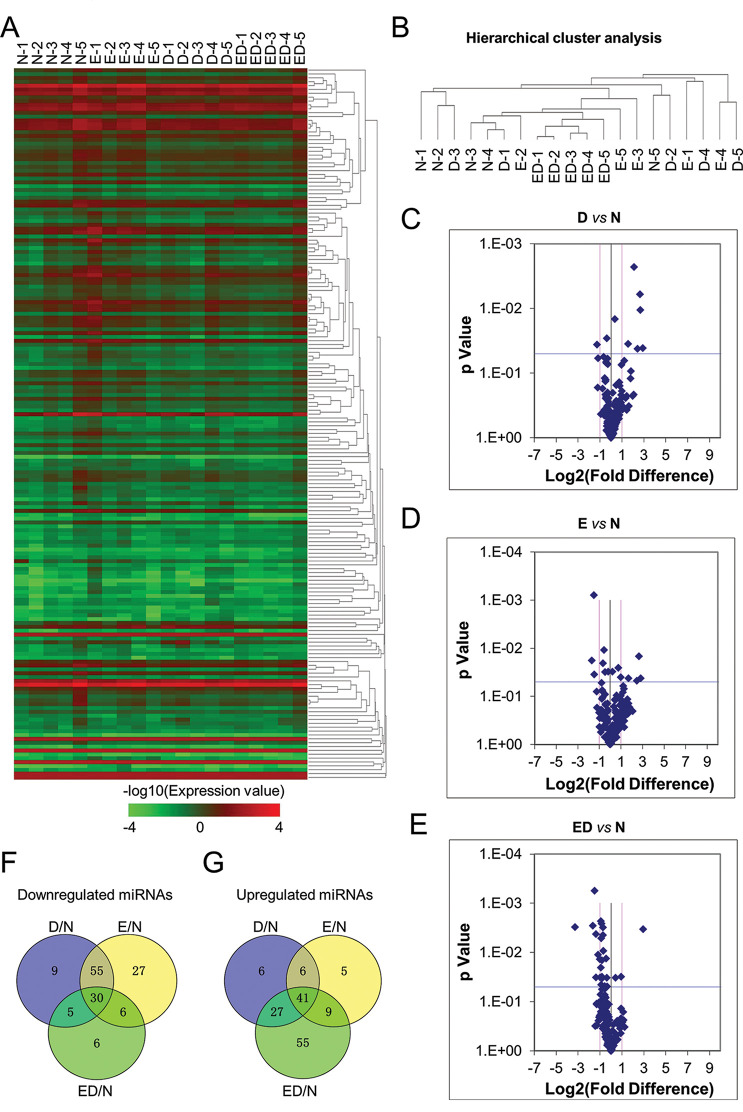
Comparation of plasma miRNA expression profile among patients and healthy donors. **A** Heatmap assay of the differentially expressed miRNAs in plasma samples of healthy donors (N-1, N-2, N-3, N-4, N-5), patients with essential hypertension (E-1, E-2, E-3, E-4, E-5), type 2 diabetes (D-1, D-2, D-3, D-4, D-5), patients with essential hypertension and type 2 diabetes mellitus (ED-1, ED-2, ED-3, ED-4, ED-5). **B** Hierarchical cluster analysis of plasma miRNA expression profile among patients (E, D, ED) and healthy donors (N). **C-E** The scatter plots illustration of differentially expressed miRNAs between the D and N groups (**C**), E and N groups (**D**), and ED and N groups (**E**). **F-G** Venn Map analysis of the distributions of the differentially downregulated (**F**) or upregulated (**G**) miRNAs in plasma samples among the indicated groups (N, E, D, and ED).

**Figure S2:**
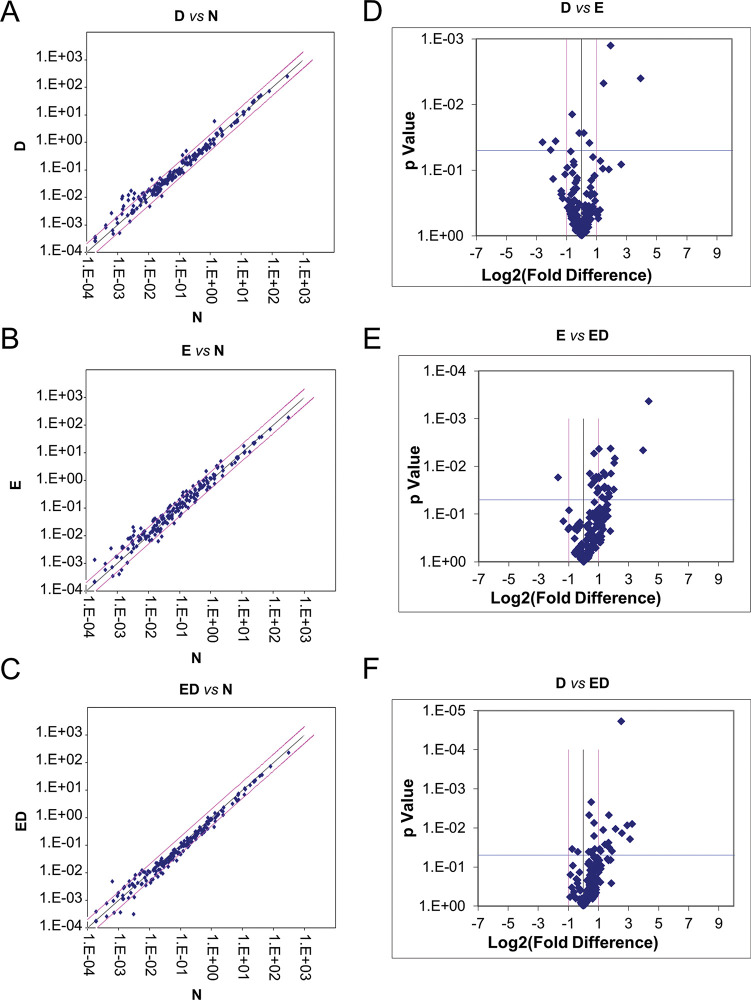
Comparation of plasma miRNA expression profiling among patients and healthy donors. **A-C** Scatter plot illustration of serum miRNAs between the D and N groups (**A**), E and N groups (**B**), and ED and N groups (**C**). **D-F** Scatter plot illustration of serum miRNAs between the D and E groups (**D**), E and ED groups (**E**), and D and ED groups (**F**).

For the purpose of separating the differentially expressed miRNA spectra and identifying potentially novel biomarkers with clinical significance, a miRNA array containing 192 human miRNAs was performed to evaluate the similarities and differences of plasma miRNA expression profiles among the patients with EH (E group), T2DM (D group), EH and T2DM (ED group) together with unrelated healthy control group (N). Compared with the N group, a subset of 31 miRNAs showed a significantly different expression profile in the ED group, and in particular, the top 10 upregulated or downregulated ones (Figure 2A, Supplementary Table S3-S4, Table S6). In the hierarchical cluster analysis, we found all the ED samples were clustered together and displayed a more distinguishable miRNA expression pattern, whereas those in the other three groups did not exhibit visible distinctions (Figure 2B). Simultaneously, as shown by the scatter plots, the majority of the 192 miRNAs between E/N, D/N, an ED/N were located in regions with fold change lower than 1 (log_2_FC<1) (Figure 2C-2E, Figure S2A-S2F, Supplementary Table S6). Furthermore, Venn Map diagrams showed that a cohort of 30 and 41 differentially downregulated and upregulated miRNAs, respectively, were collectively enriched among the four indicated groups (Figure 2F-2G).

### Verification of candidate serum miRNAs involved in patients with EH and T2DM

**Figure 3:**
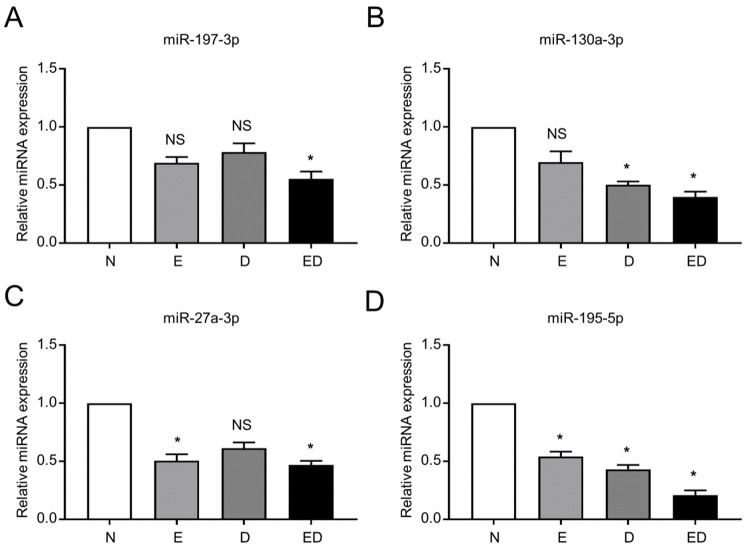
Plasma miR-195-5p expression was downregulated in the patients. **A-D** Validation of plasma levels of significantly downregulated miRNAs, including miR-197-5p (**A**), miR-130a-5p (**B**), miR-27a-5p (**C**), and miR-195-5p (**D**) by miRNA chip (**A**) and qRT-PCR. U6 was used as an internal control. All data are shown as the mean±SEM (n=5), *p<0.05; NS, not significant.

**Figure S3:**
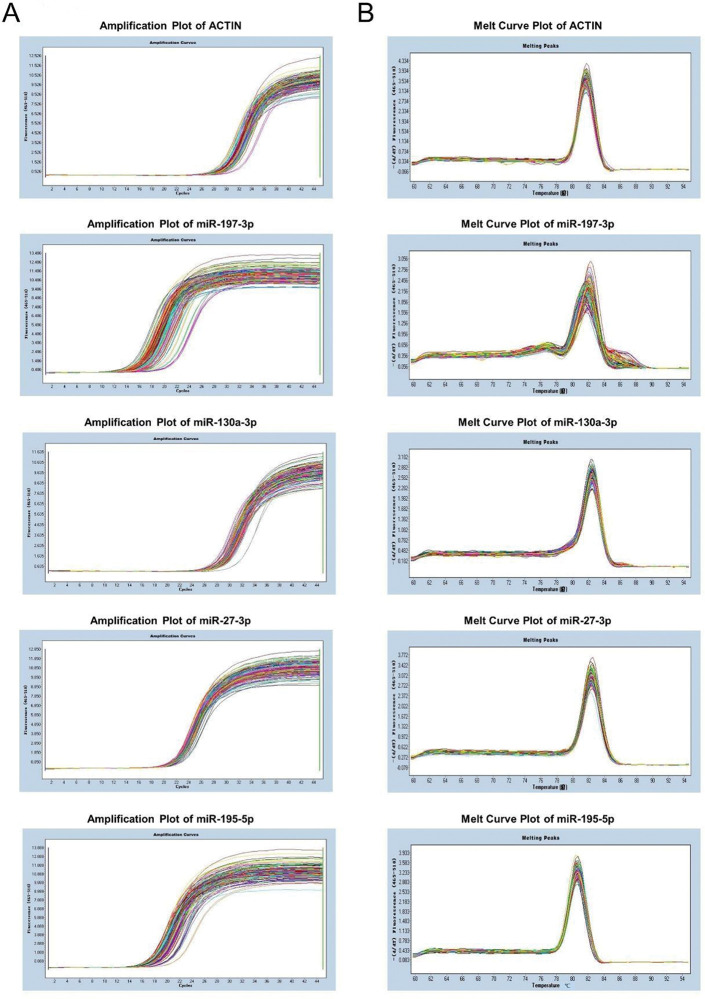
Identification of differentially expressed miRNAs among patients and healthy donors. **A-B** Amplification plot (**A**) and melting curve plot (**B**) of ACTIN, miR-197-3p, miR-130a-3p, miR-27-3p, and miR-195-5p.

Having preliminarily verified the serum miRNA expression pattern, we further attempted to explore the feasibility of candidate miRNAs as novel diagnostic indicators for patients with EH and T2DM. Of the 71 differentially regulated plasma miRNAs, only the four most downregulated ones, including miR-197-5p, miR-130a-5p, miR-27a-, and miR-195-5p, with rigorous variation screening (log_2_FC>2) were candidates for the significant discriminate of the ED and N groups (Figure 2F-2G, Supplementary Table S3-S4). Along with the miRNA chip, qRT-qPCR analysis was conducted to further identify the expressions of the aforementioned miRNAs among the four groups. Consistent with the trend in the miRNA chip, the expression levels of all candidate miRNAs were downregulated in the ED group (Figure 3A-3D, Figure S3A-S3B). For instance, miR-197-3p was only differentially downregulated in patients with ED, whereas miR-130a-3p and miR-27a-3p revealed the opposite trend in E and D groups, respectively (Figure 3A-3C). However, to distinguish from the other candidate miRNAs (miR-197-3p, miR-130a-3p, and miR-27a-3p), only miR-195-5p was also downregulated in the E and D groups. Above all, miR-195-5p expression in patients with EH and T2DM (the ED group) displayed a sharper decrease than that in the E and D groups (Figure 3D). Taken together, our data indicated that plasma miR-195-3p could potentially serve as a novel diagnostic biomarker for patients with EH and T2DM.

### DRD1 as a downstream target gene of miR-195-5p

**Figure 4:**
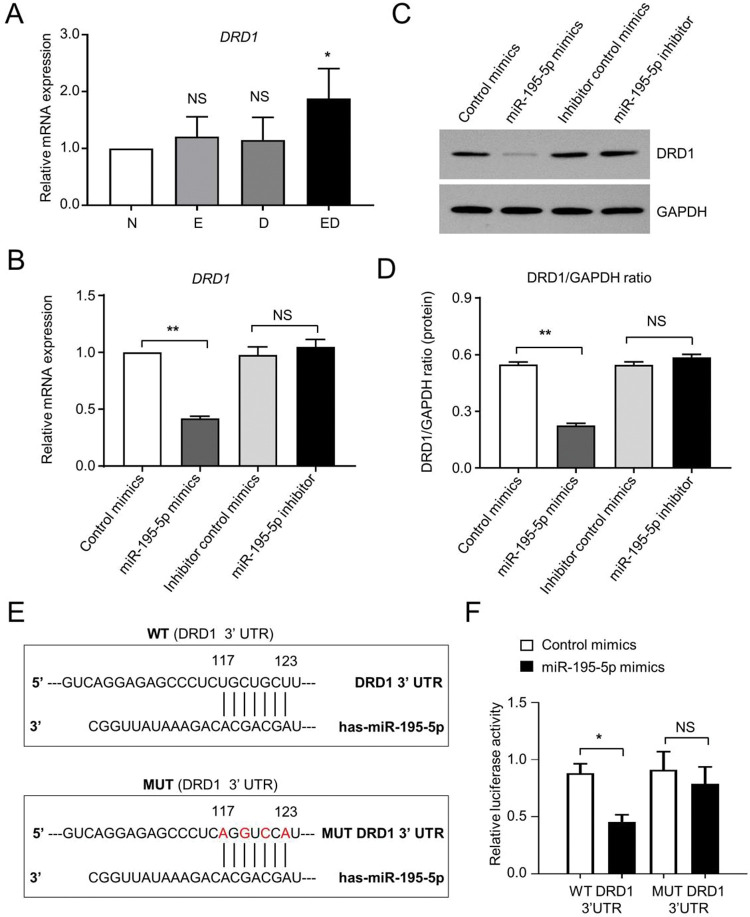
DRD1 functions as the direct inhibitory target of miR-195-5p. **A** Validation of plasma levels of DRD1 by qRT-PCR. **B** Fold change of *DRD1* mRNA expression in HEK-293T cells after transfection of miR-195-5p mimic or miR-195-5p inhibitor for 48h. Control mimics and inhibitor control mimics served as negative controls of miR-195-5p mimics and miR-195-5p inhibitors, respectively. **C-D** Expression levels of DRD1 proteins in the aforementioned four groups were quantified by western-blot assay (**C**), and gray-scale scanning with ImageJ software (**D**). GAPDH was used as an internal control. All data are shown as the mean±SEM (n=3), *p<0.05. **E** Putative wildtype (WT) and mutant type (MUT) sequences of miR-195-5p binding sites in the DRD1 3′ UTR. **F** Luciferase reporter assay of the inhibitory activity of miR-195-5p upon transfection with DRD1 3′-UTR and DRD1-mut 3'-UTR and/or miR-195-5p mimic or control mimic in in HEK-293T cells for 24h. All data are shown as the mean±SEM (n=3), *p<0.05; NS, not significant.

To further explore the molecular mechanism of serum miR-195-5p in patients with EH with T2DM, we took advantage of multiple bioinformatic platforms for target prediction, including the miRDB, TargetScanHuman, and TargetMiner. By overlapping with the Venn Map, we found that DRD1, which was the most abundant dopamine receptor in the central nervous system, showed a preferably negative correlation with miR-195-5p. Hence, we detected the expression of circulating *DRD1* among the indicated groups by utilizing qRT-PCR analysis, and found that the ED group had higher level of *DRD1* mRNA expression did the other groups (N, E, and D), which preliminarily indicated the potential of *DRD1* as the candidate target of miR-195-5p in the ED group (Figure 4A). Thereafter, we delivered the over-expressive and inhibitory mimics of miR-195-5p, and the corresponding control mimics into HEK-293T cells through transfection. After 48h post-transfection, total RNAs and proteins were isolated from HEK-293T cells for DRD1 expression detection. Quantitative analysis by qRT-PCR and western blotting showed that miR-195-5p overexpression significantly suppressed DRD1 expression and the suppressive effect was eliminated by miR-195-5p inhibition (Figures 4B-4D).

**Figure S4:**
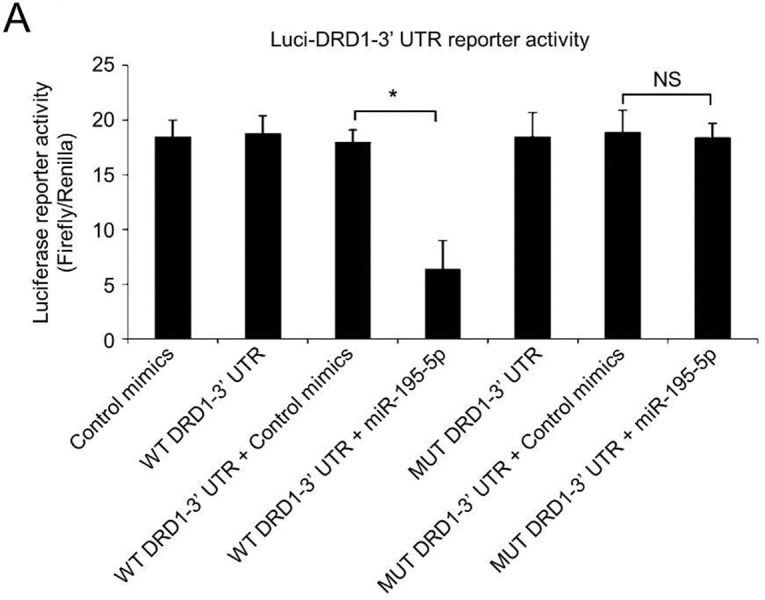
Luciferase reporter assay to investigate the binding of miR-195-5p to the DRD1 3′-UTR. **A** Luciferase reporter activity indicating the inhibitory effect of miR-195-5p on DRD1 in HEK-293T cells transfected with DRD1-3′-UTR and DRD1-mut 3'-UTR (MUT DRD1-3′ UTR) and/or miR-195-5p mimic or control mimic for 24h. All values are shown as firefly/renilla ratio. All data are shown as the mean±SEM (n=3), *p<0.05; NS, not significant.

To assess whether miR-195-5p directly targets *DRD1* to result in dysfunction in patients with EH and T2DM, the putative binding site for miR-195-5p in the 3′-UTR of *DRD1* was predicted using TargetScan (Figure 4E). Then, the luciferase-based reporter constructs either with the wild type (WT) or mutant (MUT) binding site were respectively co-transfected with miR-195-5p or Control mimics into HEK-293T cells to test their response to ectopic expression of miR-195-5p (Figure 4F, Figure S4A). Strikingly, the relative luciferase activity in the WT+miR-195-5p mimic group was lower than that in the WT DRD1 3′ UTR+Control mimic group (WT DRD1 3′ UTR+miR-195-5p mimic *vs.* WT DRD1 3′ UTR+Control mimic, 17.96±2.84 *vs* 9.10±1.75, *p*=0.0388), whereas there was minimal difference between the MUT+miR-195-5p mimic group and MUT+miR-195-5p mimic group (Figure 4E, Figure S4A). Taken together, DRD1 functioned as a direct downstream target of miR-195-5p through the abovementioned binding site of miR-195-5p on the 3′-UTR.

### SNP mutation identification in the DRD1 3′-UTR and diagnostic arability of serum miR-195-5p

**Figure 5:**
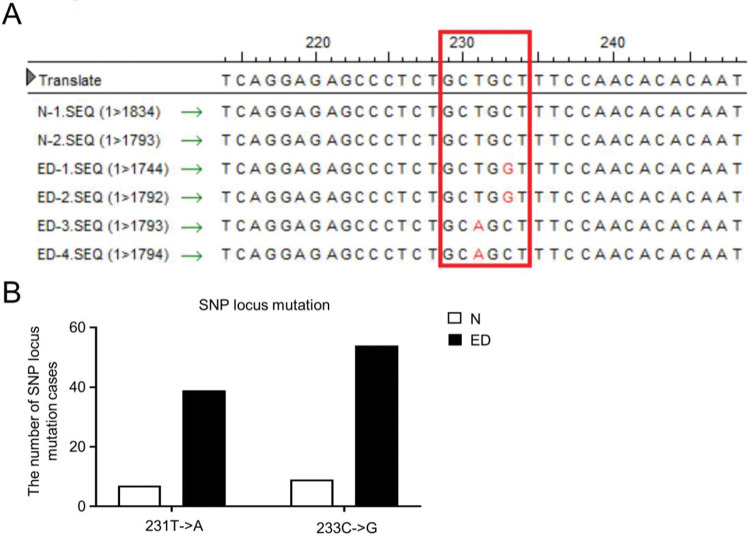
ED patients exhibited miR-195-5p binding SNP locus mutation in the 3′-UTR of DRD1. **A** Two miR-195-5p binding SNP locus mutations (231T->A, 233C->G) were identified in the 3′-UTR of DRD1 in patients with essential hypertension and type 2 diabetes mellitus (ED group). **B** Number of patients with SNP locus mutation in the ED and N groups.

**Figure S5:**
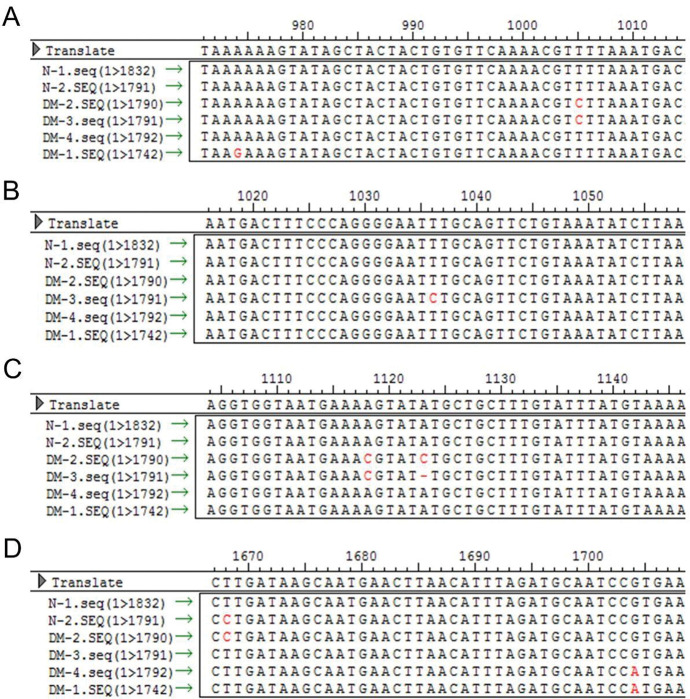
miRNA-binding SNP locus mutation analyses in the 3′-UTR of DRD1 in the ED group. **A** Analyses of miRNA-binding SNPs in other regions of the 3′-UTR of DRD1 in healthy donors (N-1, N-2) and ED groups with essential hypertension and type 2 diabetes mellitus (DM-1, DM-2, DM-3, DM-4), such as 971-1014 (**A**), 1016-1058 (**B**), 1104-1146, (**C**) and 1667-1708 (**D**).

To further validate the SNPs in the 3′-UTR of DRD1 and miR-195-5p-binding regions in patients with EH with T2DM in Chinese individuals, a case control study was performed. For the purpose, we identified two SNPs in the miR-195-5p-binding region of the DRD1 3′-UTR in a group of 200 Chinese individuals by sequencing (Figure 5A). We distinguished two common SNPs, 231T-A and 233C-G, in the DRD1 3′-UTR (based on allele frequencies) in 100 ED patients and 100 control individuals (Figure 5A, Figure S5A-S5D, Supplementary Table S5). The allele frequency of both the 231A and 233G loci in the ED group was higher than that in the N group (231A: ED *vs.* N, 39% *vs.* 7%; 233G: ED *vs.* N, 54% *vs.* 9%; Figure 5B). Collectively, these data showed that the frequencies of these two polymorphisms were closely related to EH with T2DM (*p*<0.05).

**Figure 6:**
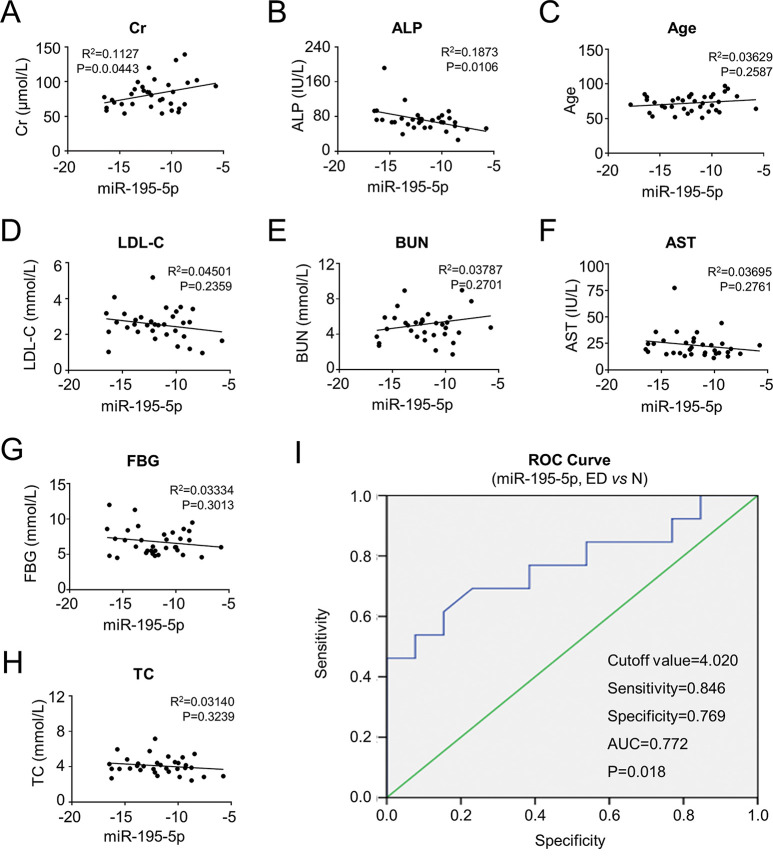
Relevance of miR-195-5p with multiple clinicopathological parameters. **A-B** The relevance of miR-195-5p with positively related ED-associated clinicopathological parameters, including the Cr (**A**) and ALP (**B**). **C** Relevance of miR-195-5p with age. **D-H** MiR-195-5p showed no significant difference with other ED-associated clinicopathological parameters, including LDL-C (**D**), BUN (**E**), AST (**F**), FBG (**G**), and TC (**H**). **I** ROC analysis of miR-195-5p between N and ED groups. Cut-off value=4.020, Sensitivity=0.846, Specificity=0.769, AUC=0.772, *p*=0.018.

**Figure S6:**
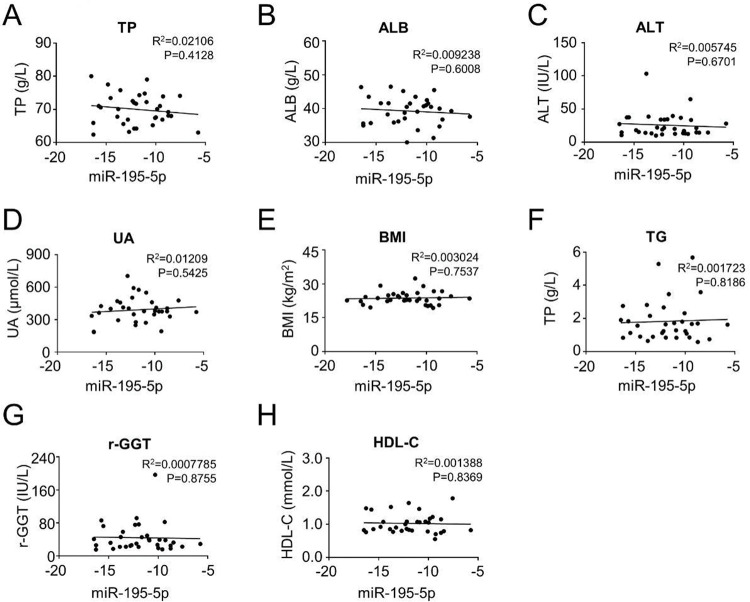
The relevance of miR-195-5p with multiple clinicopathological parameters. **A-H** MiR-195-5p showed no significant difference with other ED-associated clinicopathological parameters, including the TP (**A**), ALB (**B**), ALT (**C**), UA (**D**), BMI (**E**), TG (**F**), r-GGT (**G**), and HDL-C (**H**) values.

Additionally, to further verify the potentially clinical significance and correlation of serum miR-195-5p in diagnosis of patients with EH and T2DM, we conducted a correlation analyses with multiple clinicopathologic indexes. Of them, miR-195-5p showed a positive correlation with Cr and ALP (*p*<0.05) whereas weak or no correlation occurred with other parameters (Figures 6A-6H, Figures S6A-6H). Moreover, to further elucidate the clinical significance of miR-195-5p in patients with EH and T2DM, conducted an ROC analysis of miR-195-5p between the N group and patients with EH. We found that the area under the curve (AUC) was 0.772, and the sensitivity and specificity was 0.846 and 0.769 under the cut-off level 4.020, respectively (Figure 6I). Taken together, our data indicated that circulating miR-195-5p in peripheral blood held the potential of serving as a novel biomarker for EH and T2DM diagnosis.

## DISCUSSION

For decades, researchers have focused on the potential etiology and pathogenesis of EH or T2DM; however, novel non-invasive biomarkers and candidate targets, including miRNAs for the diagnosis and treatment of patients with EH and T2DM are still unclear (1,29). In this study, we identified serum miR-195-5p as a pivotal factor in patients with EH and T2DM. Interestingly, miR-195-5p expression displayed more remarkable downregulation than that in patients with either EH or T2DM, which indicated the potential of miR-195-5p to serve as a novel biomarker and therapeutic target for EH with T2DM. Furthermore, with the aid of the luciferase-based reporter assay and SNP analyses, we identified *DRD1* as a direct target of miR-195-5p in patients with EH and T2DM.

Hypertension is a complicated multifactorial disease attributable to dysregulation of angiogenesis and vascular smooth muscle, myocardial hypertrophy, activation of the renin-angiotensin-aldosterone system and platelet functional impairment. Because of the development and progression of EH and T2DM, morbidity and mortality in cardiovascular diseases collectively deteriorated as well. Furthermore, hypertension is acknowledged as being involved with more than two-thirds of patients with T2DM (3). Despite notable progress for the diagnosis and management of patients with EH or T2DM according to the guidelines, the development of more accurate clinical therapeutics is far from satisfactory. This is mainly caused by the deficiency in novel non-invasive biomarkers and therapeutic target identification.

MicroRNAs are evolutionarily conserved single-stranded transcripts of hairpin structures with 21-23 nucleotides throughout the genome, which are involved in multifaceted physiological and pathological processes by regulating gene-expression post-transcriptionally, and serving as promising candidates for biomarker and targeted therapy development (30). Numerous functional studies have indicated the dysregulation and potential clinical significance of miRNAs in EH (*e.g.*, miR-92a, miR-31a-5p) and T2DM (*e.g.*, miR-15a, miR-223, miR-375, miR-30d) patients (13,14). For instance, serum miR-92a holds potential to function as a non-invasive marker of atherosclerosis in hypertension, whereas miR-31a-5p and miR-184 are involved in hypertension and glucose metabolism by bidirectional regulation of arterial smooth muscle cell and β cell function, respectively (31,32). Herein, with the aid of an miRNA qRT-PCR array, serum miR-195-5p was identified as a novel non-invasive diagnostic biomarker in patients with EH and T2DM, which was identified as a tumor suppressor and an indicator of poor prognosis in tumor progression (33). Thus, in this study we identified the novel dysregulation and malfunction of miR-195-5p in Chinese individuals with T2DM and EH, which collectively indicated the multidimensional functionality and pathogenesis of miRNAs. Therefore, studies on the characteristics of miRNAs in the etiology and pathogenesis of patient with EH and T2DM should be undertaken systematically and meticulously.

Dopamine is an important neurotransmitter that regulates diverse physiological processes including behavior, hormone synthesis and release, blood pressure, and transmembrane ion transport. Dopamine receptor D1 (DRD1) belongs to the superfamily of G-protein-coupled receptors (GPCRs), and is classified by structure and pharmacology, which is acknowledged as the most important DR subtype for sodium reuptake and renal sodium excretion, while dysfunction of DRD1 commonly results in chronic hypertension in patients with EH (34). Herein, we further illuminated the negative regulation of *DRD1* by miR-195-5p via miRNA-mediated cleavage and translational repression of target genes. Furthermore, we identified two SNPs, 231T-A and 233C-G, in the miR-195-5p-binding sites in the DRD1 3′-UTR. Distinguished from those in the health donors, frequencies of the 231A and 233G alleles were much higher in the patients with EH and T2DM, which subsequently altered the binding affinity of miR-195-5p toward the 3′-UTR and deregulated the post-transcriptional regulation of *DRD1*. Taken together, our research revealed that serum miR-195-5p exhibits clinical significance with respect to non-invasive diagnosis and interventional therapeutics and provides insights into the pathogenesis of EH with T2DM.

## CONCLUSIONS

Overall, in this study we identified serum miR-195-5p as a novel non-invasive biomarker for clinical diagnosis and interventional therapeutics for EH with concomitant T2DM that functions by by directly suppressing DRD1 expression. Taken together, our findings further elucidated the pathogenesis of EH with T2DM and provided promising candidates for the development of novel targeted agents.

## AUTHOR CONTRIBUTIONS

Hu Y, Li Q and Zhang L designed and performed the experiments, were responsible for the data collection and assembly, and manuscript writing. Zhong L, Gu M, He B, Qu Q, Lao Y and Gu K helped with collection and assembly of data. Zhang L, Zheng B and Yang H were responsible for the conception and design, data analysis and interpretation, manuscript writing and revision, approval of the final version of the manuscript. The final version of the manuscript has been read and approved by all the authors.

## APPENDIX

**Table S1:** General information of representative groups (E, D, ED) and healthy donors (N).

Group	NO.	Gender	Age (Year)	Weight (kg)	Height (m)	BMI	TP (g/L)	ALB (g/L)	Liver Function (IU/L)				Renal Function		UA (μmol /L)	FBG (mmol/L)	Blood Lipid (mmol/L)			
									ALT	AST	ALP	r-GGT	Cr (μmol/L)	BUN (mmol/L)			TC	TG	HLC-C	LDL-C
N	1	F	93	50	1.70	17.3	70.9	43.7	17.6	20.5	33.6	15.9	89.4	4.47	498	5.4	4.48	1.15	1.23	3.03
	2	M	43	69	1.72	23.3	75.3	47.7	39.8	22.9	68.3	39.4	85.2	2.98	489.5	4.3	1.34	1.59	1.04	3.3
	3	M	61	63	1.72	21.1	70.9	43.7	17.6	20.5	33.6	15.9	89.4	4.47	498	5.4	4.48	1.15	1.23	3.03
	4	F	67	60	1.48	27.4	77.3	41.0	22.5	25.5	91.7	83.7	63.5	4.45	354.9	4.6				
	5	M	96	45	1.55	18.7	59.4	31.3	17	26	21	112	123.7	10.43	389.6	4.9	2.98	0.53	1.52	1.41
	6	M	79	50	1.60	19.5	67.7	38.6	10.4	21.7	52.9	18.9	124.3	5.03	375.9	4.7	4.08	0.86	1.54	2.4
E	1	M	82	60	1.63	22.6	72.0	38.8	11.7	14.7	65.8	30.1	120.1	5.64	592.1	5.1	3.35	1.23	0.86	2.21
	2	F	73	52	1.63	19.6	70.6	35.7	37.4	35.9	192	72.5	69.9	4.6	424.6	4.5	3.74	1.12	0.85	2.68
	3	F	81	53	1.60	20.7	62.4	35.0	14.8	24.6	93.7	26	62.2	2.96	184.4	4.8	3.75	0.83	1.48	2.15
	4	M	76	67	1.65	24.6	66.9	36.2	9.9	13.1	73	24.7	92.3	5.32	703.2	5.2				
	5	F	85	55	1.55	22.9	71.7	40.5	21.6	26.7	70	22.2	63.8	4.22	248.5	4.8	4.43	1.64	1.64	2.56
	6	F	51	54	1.55	22.5	72.2	39.1	20.1	14.2	71.5	40.2	54.1	2.16	271.4	5.9	5.15	1.28	1.46	3.5
	7	M	75	69	1.74	22.8	74.3	41.5	34.5	35.5	54.3	45.5	103.3	6.26	575	4.9	4.19	3.46	0.81	2.52
	8	M	82	75	1.75	24.5	74.1	39.3	14.5	15.2	50.8	22.6	102	7.73	476.9	4.6	2.83	0.74	1.78	0.97
	9	F	81	53	1.53	22.6	64.2	37.1	19	21	75.1	91.7	84.5	5.5	349.3	6.1	7.16	2.66	1.08	5.18
	10	F	74	47	1.56	19.3	66.9	31.3	64.8	44.2	92.2	82.3	56	1.71	192.2	4.9	3.85	1.66	0.55	2.65
D	1	M	85	60	1.64	22.3	75.5	49.2	18.7	17.1	73.4	18.8	78.4	6.79	513.8	7.9	4.8	1.32	1.17	3.16
	2	M	68	66	1.67	23.7	80.0	46.4	27.3	19.4	93.1	40.9	77.7	3.72	334.9	8.6	4.29	1.9	0.82	3.18
	3	M	76	72	1.70	24.9	77.5		18.1	27.6	66.9	31.9	67.5	5.83		7	4.82	1.56	0.92	3.15
	4	M	62	51	1.58	20.4	70.1	41.6	103.3	77.4	39.5	45.9	67.7	5.27	294.5	6.1	4.35	0.64	1.52	2.81
	5	M	69	67	1.72	22.6	71.0	40.5	12.4	16	45.4	16	131.1	5.92	375.3	7.2	5.06	0.85	1.22	3.53
	6	F	61	60	1.55	25.0	67.6	42.5	17	18.3	82.9	19.2	58.3	4.72	379.7	6	4.41	1.23	1.05	3.28
	7	M	81	72	1.73	24.1	64.2	30	34.1	29.8	74.2	76.4	80.6	3.31	276.9	5.5	2.95	0.84	0.83	1.76
ED	1	M	72	75	1.70	26.0	79.0	33.6	12.6	16.2	71.4	37.8	74.8	5.25	549.6	7.1	3.43	0.83	1.06	2
	2	M	53	66	1.67	24.5	71.0	43.5	37.3	24.7	72.6	86.1	73.5	5.89	362.4	7.2	5.96	1.83	1.44	4.08
	3	M	67	63	1.68	22.3	67.8	38.3	14.1	16	75.4	22.4	82.2	8.95	470.9	11.3	4.1	2.81	0.85	2.56
	4	F	66	64	1.48	29.2	73.4	41.6	15.4	14.8	67	17.4	54.1	7.2	399.9	8.4	3.79	0.9	1.07	2.39
	5	M	52	70	1.75	22.9	75.8	46.5	38.5	36	118	58.4	89	5.01	457.7	9	3.62	2.15	0.77	2.15
	6	M	60	90	1.76	29.1	65.2	40.7	36.8	23.3	77	196.5	95.2	5.1	460.2	8.1	4.52	1.8	1.08	3
	7	M	57	75	1.70	26.0	72.5	45.2	34	19.1	83.1	75.6	84.8	5.22	503	5.5	3.73	1.12	1	2.72
	8	M	59	81	1.74	26.8	73.9	45.5	33.4	24.7	76.2	38.2	60.9	3	375	8.6	4.16	5.67	0.7	1.89
	9	M	64	68	1.68	24.1	63.2	38.1	22	25.5	54.8	26.2	87.1	3.71	403.6	5.5	5.76	5.28	0.8	2.52
	10	M	93	81	1.74	26.8	68.0	36.8	14.3	19.8	26.4	32.8	98.4	8.98	329.2	9.5	5.44	3.58	0.79	3.42
	11	M	85	55	1.65	20.2	70.1	41.5	13.2	15.4	66.4	39.2	85.2	5.32	410.2	5.6	2.83	1.08	1.17	1.33
	12	M	67	83	1.60	32.4	74.8	43.5	39.5	23.8	55.3	49.2	73.2	3.89	377.6	7.8	3.8	1.72	1.07	2.56
	13	M	97	68	1.70	23.5	68.1	40	14.8	12.7	66.7	18.4	139.2	12.58	404.7	7	2.43	0.58	1.15	1.2
	14	F	77	64	1.62	23.1	65.9	35.6	10.5	17.1	72.6	15.4	58.3ss	2.7	188.8	12	2.68	2.75	0.76	1.03
	15	M	79	78	1.75	25.5	65.6	35.9	13.3	15.8	62.3	22.5	99.4	4.4	413.2	7	4.06	0.89	0.9	2.96
	16	F	81	54	1.62	20.6	67.4	41	13.6	11.2	67.3	27.1	68.3	4.02	347.9	6	3.75	2.31	0.78	2.21
	17	M	89	56	1.65	20.6	69.2	34.7	21.3	16.6	58.2	25	67.4	4.18	376.1	8.3	3.88	1.69	0.74	2.69
	18	M	64	61	1.61	23.5	63.0	37.6	28	23.2	52.8	29	93.6	4.76	369.9	6	2.92	1.62	0.82	1.65

**Table S2:** Primer sequences for the amplification of candidate miRNAs.

miR name (human)	microRNA target sequence
hsa-miR-652-3p	AAUGGCGCCACUAGGGUUGUG
hsa-miR-502-3p	AAUGCACCUGGGCAAGGAUUCA
hsa-miR-181a-5p	AACAUUCAACGCUGUCGGUGAGU
hsa-miR-339-3p	UGAGCGCCUCGACGACAGAGCCG
hsa-miR-221-3p	AGCUACAUUGUCUGCUGGGUUUC
hsa-miR-409-3p	GAAUGUUGCUCGGUGAACCCCU
hsa-let-7f-5p	UGAGGUAGUAGAUUGUAUAGUU
hsa-miR-154-5p	UAGGUUAUCCGUGUUGCCUUCG
hsa-miR-27b-3p	UUCACAGUGGCUAAGUUCUGC
hsa-miR-155-5p	UUAAUGCUAAUCGUGAUAGGGGU
hsa-miR-374b-5p	AUAUAAUACAACCUGCUAAGUG
hsa-miR-140-3p	UACCACAGGGUAGAACCACGG
hsa-miR-93-5p	CAAAGUGCUGUUCGUGCAGGUAG
hsa-miR-92b-3p	UAUUGCACUCGUCCCGGCCUCC
hsa-miR-200a-3p	UAACACUGUCUGGUAACGAUGU
hsa-miR-505-3p	CGUCAACACUUGCUGGUUUCCU
hsa-miR-23b-3p	AUCACAUUGCCAGGGAUUUCC
hsa-miR-484	UCAGGCUCAGUCCCCUCCCGAU
hsa-miR-141-3p	UAACACUGUCUGGUAAAGAUGG
hsa-miR-10a-5p	UACCCUGUAGAUCCGAAUUUGUG
hsa-miR-106a-5p	AAAAGUGCUUACAGUGCAGGUAG
hsa-miR-27a-3p	UUCACAGUGGCUAAGUUCCGC
hsa-miR-652-3p	AAUGGCGCCACUAGGGUUGUG
hsa-miR-502-3p	AAUGCACCUGGGCAAGGAUUCA
hsa-miR-181a-5p	AACAUUCAACGCUGUCGGUGAGU
hsa-miR-339-3p	UGAGCGCCUCGACGACAGAGCCG
hsa-miR-221-3p	AGCUACAUUGUCUGCUGGGUUUC
hsa-miR-409-3p	GAAUGUUGCUCGGUGAACCCCU
hsa-let-7f-5p	UGAGGUAGUAGAUUGUAUAGUU
hsa-miR-154-5p	UAGGUUAUCCGUGUUGCCUUCG
hsa-miR-27b-3p	UUCACAGUGGCUAAGUUCUGC
hsa-miR-155-5p	UUAAUGCUAAUCGUGAUAGGGGU
hsa-miR-374b-5p	AUAUAAUACAACCUGCUAAGUG
hsa-miR-140-3p	UACCACAGGGUAGAACCACGG
hsa-miR-93-5p	CAAAGUGCUGUUCGUGCAGGUAG
hsa-miR-92b-3p	UAUUGCACUCGUCCCGGCCUCC
hsa-miR-200a-3p	UAACACUGUCUGGUAACGAUGU
hsa-miR-505-3p	CGUCAACACUUGCUGGUUUCCU
hsa-miR-23b-3p	AUCACAUUGCCAGGGAUUUCC
hsa-miR-484	UCAGGCUCAGUCCCCUCCCGAU
hsa-miR-141-3p	UAACACUGUCUGGUAAAGAUGG
hsa-miR-10a-5p	UACCCUGUAGAUCCGAAUUUGUG
hsa-miR-106a-5p	AAAAGUGCUUACAGUGCAGGUAG
hsa-miR-27a-3p	UUCACAGUGGCUAAGUUCCGC
hsa-miR-145-5p	GUCCAGUUUUCCCAGGAAUCCCU
hsa-miR-1	UGGAAUGUAAAGAAGUAUGUAU
hsa-miR-185-5p	UGGAGAGAAAGGCAGUUCCUGA
hsa-miR-382-5p	GAAGUUGUUCGUGGUGGAUUCG
hsa-miR-486-5p	UCCUGUACUGAGCUGCCCCGAG
hsa-miR-32-5p	UAUUGCACAUUACUAAGUUGCA
hsa-miR-26a-5p	UUCAAGUAAUCCAGGAUAGGCU
hsa-miR-133b	UUUGGUCCCCUUCAACCAGCUG
hsa-miR-143-3p	UGAGAUGAAGCACUGUAGCUC
hsa-let-7d-5p	AGAGGUAGUAGGUUGCAUAGUU
hsa-miR-30a-5p	UGUAAACAUCCUCGACUGGAAG
hsa-miR-133a	UUUGGUCCCCUUCAACCAGCUG
hsa-miR-222-3p	AGCUACAUCUGGCUACUGGGU
hsa-miR-20b-5p	CAAAGUGCUCAUAGUGCAGGUAG
hsa-miR-342-3p	UCUCACACAGAAAUCGCACCCGU
hsa-miR-328	CUGGCCCUCUCUGCCCUUCCGU
hsa-miR-324-5p	CGCAUCCCCUAGGGCAUUGGUGU
hsa-miR-532-3p	CCUCCCACACCCAAGGCUUGCA
hsa-miR-15a-5p	UAGCAGCACAUAAUGGUUUGUG
hsa-let-7e-5p	UGAGGUAGGAGGUUGUAUAGUU
hsa-miR-532-5p	CAUGCCUUGAGUGUAGGACCGU
hsa-miR-145-5p	GUCCAGUUUUCCCAGGAAUCCCU
hsa-miR-1	UGGAAUGUAAAGAAGUAUGUAU
hsa-miR-185-5p	UGGAGAGAAAGGCAGUUCCUGA
hsa-miR-382-5p	GAAGUUGUUCGUGGUGGAUUCG
hsa-miR-486-5p	UCCUGUACUGAGCUGCCCCGAG
hsa-miR-32-5p	UAUUGCACAUUACUAAGUUGCA
hsa-miR-26a-5p	UUCAAGUAAUCCAGGAUAGGCU
hsa-miR-133b	UUUGGUCCCCUUCAACCAGCUG
hsa-miR-143-3p	UGAGAUGAAGCACUGUAGCUC
hsa-let-7d-5p	AGAGGUAGUAGGUUGCAUAGUU
hsa-miR-30a-5p	UGUAAACAUCCUCGACUGGAAG
hsa-miR-133a	UUUGGUCCCCUUCAACCAGCUG
hsa-miR-222-3p	AGCUACAUCUGGCUACUGGGU
hsa-miR-20b-5p	CAAAGUGCUCAUAGUGCAGGUAG
hsa-miR-342-3p	UCUCACACAGAAAUCGCACCCGU
hsa-miR-328	CUGGCCCUCUCUGCCCUUCCGU
hsa-miR-324-5p	CGCAUCCCCUAGGGCAUUGGUGU
hsa-miR-532-3p	CCUCCCACACCCAAGGCUUGCA
hsa-miR-15a-5p	UAGCAGCACAUAAUGGUUUGUG
hsa-let-7e-5p	UGAGGUAGGAGGUUGUAUAGUU
hsa-miR-532-5p	CAUGCCUUGAGUGUAGGACCGU
hsa-miR-139-5p	UCUACAGUGCACGUGUCUCCAGU
hsa-miR-194-5p	UGUAACAGCAACUCCAUGUGGA
hsa-miR-375	UUUGUUCGUUCGGCUCGCGUGA
hsa-miR-660-5p	UACCCAUUGCAUAUCGGAGUUG
hsa-miR-451a	AAACCGUUACCAUUACUGAGUU
hsa-miR-574-3p	CACGCUCAUGCACACACCCACA
hsa-let-7a-5p	UGAGGUAGUAGGUUGUAUAGUU
hsa-miR-551b-3p	GCGACCCAUACUUGGUUUCAG
hsa-miR-128	UCACAGUGAACCGGUCUCUUU
hsa-miR-130a-3p	CAGUGCAAUGUUAAAAGGGCAU
hsa-miR-125a-5p	UCCCUGAGACCCUUUAACCUGUGA
hsa-miR-28-5p	AAGGAGCUCACAGUCUAUUGAG
hsa-miR-485-3p	GUCAUACACGGCUCUCCUCUCU
hsa-miR-497-5p	CAGCAGCACACUGUGGUUUGU
hsa-let-7b-3p	CUAUACAACCUACUGCCUUCCC
hsa-miR-425-3p	AUCGGGAAUGUCGUGUCCGCCC
hsa-miR-132-3p	UAACAGUCUACAGCCAUGGUCG
hsa-miR-25-3p	CAUUGCACUUGUCUCGGUCUGA
hsa-let-7c	UGAGGUAGUAGGUUGUAUGGUU
hsa-miR-18a-5p	UAAGGUGCAUCUAGUGCAGAUAG
hsa-miR-33a-5p	GUGCAUUGUAGUUGCAUUGCA
hsa-miR-29b-3p	UAGCACCAUUUGAAAUCAGUGUU
hsa-miR-139-5p	UCUACAGUGCACGUGUCUCCAGU
hsa-miR-194-5p	UGUAACAGCAACUCCAUGUGGA
hsa-miR-375	UUUGUUCGUUCGGCUCGCGUGA
hsa-miR-660-5p	UACCCAUUGCAUAUCGGAGUUG
hsa-miR-451a	AAACCGUUACCAUUACUGAGUU
hsa-miR-574-3p	CACGCUCAUGCACACACCCACA
hsa-let-7a-5p	UGAGGUAGUAGGUUGUAUAGUU
hsa-miR-551b-3p	GCGACCCAUACUUGGUUUCAG
hsa-miR-128	UCACAGUGAACCGGUCUCUUU
hsa-miR-130a-3p	CAGUGCAAUGUUAAAAGGGCAU
hsa-miR-125a-5p	UCCCUGAGACCCUUUAACCUGUGA
hsa-miR-28-5p	AAGGAGCUCACAGUCUAUUGAG
hsa-miR-485-3p	GUCAUACACGGCUCUCCUCUCU
hsa-miR-497-5p	CAGCAGCACACUGUGGUUUGU
hsa-let-7b-3p	CUAUACAACCUACUGCCUUCCC
hsa-miR-425-3p	AUCGGGAAUGUCGUGUCCGCCC
hsa-miR-132-3p	UAACAGUCUACAGCCAUGGUCG
hsa-miR-25-3p	CAUUGCACUUGUCUCGGUCUGA
hsa-let-7c	UGAGGUAGUAGGUUGUAUGGUU
hsa-miR-18a-5p	UAAGGUGCAUCUAGUGCAGAUAG
hsa-miR-33a-5p	GUGCAUUGUAGUUGCAUUGCA
hsa-miR-29b-3p	UAGCACCAUUUGAAAUCAGUGUU
hsa-miR-16-5p	UAGCAGCACGUAAAUAUUGGCG
hsa-miR-136-5p	ACUCCAUUUGUUUUGAUGAUGGA
hsa-let-7b-5p	UGAGGUAGUAGGUUGUGUGGUU
hsa-let-7i-3p	CUGCGCAAGCUACUGCCUUGCU
hsa-miR-152	UCAGUGCAUGACAGAACUUGG
hsa-miR-106b-5p	UAAAGUGCUGACAGUGCAGAU
hsa-miR-30c-5p	UGUAAACAUCCUACACUCUCAGC
hsa-miR-15b-3p	CGAAUCAUUAUUUGCUGCUCUA
hsa-miR-197-3p	UUCACCACCUUCUCCACCCAGC
hsa-miR-142-5p	CAUAAAGUAGAAAGCACUACU
hsa-miR-99b-5p	CACCCGUAGAACCGACCUUGCG
hsa-miR-296-5p	AGGGCCCCCCCUCAAUCCUGU
hsa-miR-30e-5p	UGUAAACAUCCUUGACUGGAAG
hsa-miR-326	CCUCUGGGCCCUUCCUCCAG
hsa-miR-146a-5p	UGAGAACUGAAUUCCAUGGGUU
cel-miR-39-3p	TCACCGGGTGTAAATCAGCTTG
hsa-miR-421	AUCAACAGACAUUAAUUGGGCGC
hsa-miR-424-5p	CAGCAGCAAUUCAUGUUUUGAA
hsa-miR-223-5p	CGUGUAUUUGACAAGCUGAGUU
hsa-miR-146b-5p	UGAGAACUGAAUUCCAUAGGCU
hsa-miR-107	AGCAGCAUUGUACAGGGCUAUGA
hsa-miR-205-5p	UCCUUCAUUCCACCGGAGUCUG
hsa-miR-16-5p	UAGCAGCACGUAAAUAUUGGCG
hsa-miR-136-5p	ACUCCAUUUGUUUUGAUGAUGGA
hsa-let-7b-5p	UGAGGUAGUAGGUUGUGUGGUU
hsa-let-7i-3p	CUGCGCAAGCUACUGCCUUGCU
hsa-miR-152	UCAGUGCAUGACAGAACUUGG
hsa-miR-106b-5p	UAAAGUGCUGACAGUGCAGAU
hsa-miR-30c-5p	UGUAAACAUCCUACACUCUCAGC
hsa-miR-15b-3p	CGAAUCAUUAUUUGCUGCUCUA
hsa-miR-197-3p	UUCACCACCUUCUCCACCCAGC
hsa-miR-142-5p	CAUAAAGUAGAAAGCACUACU
hsa-miR-99b-5p	CACCCGUAGAACCGACCUUGCG
hsa-miR-296-5p	AGGGCCCCCCCUCAAUCCUGU
hsa-miR-30e-5p	UGUAAACAUCCUUGACUGGAAG
hsa-miR-326	CCUCUGGGCCCUUCCUCCAG
hsa-miR-146a-5p	UGAGAACUGAAUUCCAUGGGUU
cel-miR-39-3p	TCACCGGGTGTAAATCAGCTTG
hsa-miR-421	AUCAACAGACAUUAAUUGGGCGC
hsa-miR-424-5p	CAGCAGCAAUUCAUGUUUUGAA
hsa-miR-223-5p	CGUGUAUUUGACAAGCUGAGUU
hsa-miR-146b-5p	UGAGAACUGAAUUCCAUAGGCU
hsa-miR-107	AGCAGCAUUGUACAGGGCUAUGA
hsa-miR-205-5p	UCCUUCAUUCCACCGGAGUCUG
hsa-miR-148b-3p	UCAGUGCAUCACAGAACUUUGU
hsa-miR-339-5p	UCCCUGUCCUCCAGGAGCUCACG
hsa-miR-20a-5p	UAAAGUGCUUAUAGUGCAGGUAG
hsa-miR-17-5p	CAAAGUGCUUACAGUGCAGGUAG
hsa-miR-500a-5p	UAAUCCUUGCUACCUGGGUGAGA
hsa-miR-30d-5p	UGUAAACAUCCCCGACUGGAAG
hsa-miR-378a-3p	ACUGGACUUGGAGUCAGAAGG
hsa-miR-186-5p	CAAAGAAUUCUCCUUUUGGGCU
hsa-miR-425-5p	AAUGACACGAUCACUCCCGUUGA
hsa-let-7i-5p	UGAGGUAGUAGUUUGUGCUGUU
hsa-miR-346	UGUCUGCCCGCAUGCCUGCCUCU
hsa-miR-26b-5p	UUCAAGUAAUUCAGGAUAGGU
hsa-miR-193b-3p	AACUGGCCCUCAAAGUCCCGCU
hsa-miR-34a-5p	UGGCAGUGUCUUAGCUGGUUGU
hsa-miR-320b	AAAAGCUGGGUUGAGAGGGCAA
hsa-miR-885-5p	UCCAUUACACUACCCUGCCUCU
hsa-miR-590-5p	GAGCUUAUUCAUAAAAGUGCAG
hsa-miR-127-3p	UCGGAUCCGUCUGAGCUUGGCU
hsa-miR-191-5p	CAACGGAAUCCCAAAAGCAGCUG
hsa-miR-208a	AUAAGACGAGCAAAAAGCUUGU
hsa-miR-99a-5p	AACCCGUAGAUCCGAUCUUGUG
hsa-miR-16-2-3p	CCAAUAUUACUGUGCUGCUUUA
hsa-miR-148b-3p	UCAGUGCAUCACAGAACUUUGU
hsa-miR-339-5p	UCCCUGUCCUCCAGGAGCUCACG
hsa-miR-20a-5p	UAAAGUGCUUAUAGUGCAGGUAG
hsa-miR-17-5p	CAAAGUGCUUACAGUGCAGGUAG
hsa-miR-500a-5p	UAAUCCUUGCUACCUGGGUGAGA
hsa-miR-30d-5p	UGUAAACAUCCCCGACUGGAAG
hsa-miR-378a-3p	ACUGGACUUGGAGUCAGAAGG
hsa-miR-186-5p	CAAAGAAUUCUCCUUUUGGGCU
hsa-miR-425-5p	AAUGACACGAUCACUCCCGUUGA
hsa-let-7i-5p	UGAGGUAGUAGUUUGUGCUGUU
hsa-miR-346	UGUCUGCCCGCAUGCCUGCCUCU
hsa-miR-26b-5p	UUCAAGUAAUUCAGGAUAGGU
hsa-miR-193b-3p	AACUGGCCCUCAAAGUCCCGCU
hsa-miR-34a-5p	UGGCAGUGUCUUAGCUGGUUGU
hsa-miR-320b	AAAAGCUGGGUUGAGAGGGCAA
hsa-miR-885-5p	UCCAUUACACUACCCUGCCUCU
hsa-miR-590-5p	GAGCUUAUUCAUAAAAGUGCAG
hsa-miR-127-3p	UCGGAUCCGUCUGAGCUUGGCU
hsa-miR-191-5p	CAACGGAAUCCCAAAAGCAGCUG
hsa-miR-208a	AUAAGACGAGCAAAAAGCUUGU
hsa-miR-99a-5p	AACCCGUAGAUCCGAUCUUGUG
hsa-miR-16-2-3p	CCAAUAUUACUGUGCUGCUUUA
hsa-miR-301a-3p	CAGUGCAAUAGUAUUGUCAAAGC
hsa-miR-140-5p	CAGUGGUUUUACCCUAUGGUAG
hsa-miR-151a-5p	UCGAGGAGCUCACAGUCUAGU
hsa-miR-130b-3p	CAGUGCAAUGAUGAAAGGGCAU
hsa-miR-122-5p	UGGAGUGUGACAAUGGUGUUUG
hsa-miR-20a-3p	ACUGCAUUAUGAGCACUUAAAG
hsa-miR-423-5p	UGAGGGGCAGAGAGCGAGACUUU
hsa-miR-629-5p	UGGGUUUACGUUGGGAGAACU
hsa-miR-101-3p	UACAGUACUGUGAUAACUGAA
hsa-miR-200c-3p	UAAUACUGCCGGGUAAUGAUGGA
hsa-miR-365a-3p	UAAUGCCCCUAAAAAUCCUUAU
hsa-miR-501-3p	AAUGCACCCGGGCAAGGAUUCU
hsa-miR-23a-3p	AUCACAUUGCCAGGGAUUUCC
hsa-miR-423-3p	AGCUCGGUCUGAGGCCCCUCAGU
hsa-miR-215	AUGACCUAUGAAUUGACAGAC
hsa-miR-376a-3p	AUCAUAGAGGAAAAUCCACGU
hsa-miR-320a	AAAAGCUGGGUUGAGAGGGCGA
hsa-miR-22-5p	AGUUCUUCAGUGGCAAGCUUUA
hsa-miR-338-3p	UCCAGCAUCAGUGAUUUUGUUG
hsa-miR-2110	UUGGGGAAACGGCCGCUGAGUG
hsa-miR-223-3p	UGUCAGUUUGUCAAAUACCCCA
hsa-miR-361-3p	UCCCCCAGGUGUGAUUCUGAUUU
hsa-miR-103a-3p	AGCAGCAUUGUACAGGGCUAUGA
hsa-miR-93-3p	ACUGCUGAGCUAGCACUUCCCG
hsa-miR-301a-3p	CAGUGCAAUAGUAUUGUCAAAGC
hsa-miR-140-5p	CAGUGGUUUUACCCUAUGGUAG
hsa-miR-151a-5p	UCGAGGAGCUCACAGUCUAGU
hsa-miR-130b-3p	CAGUGCAAUGAUGAAAGGGCAU
hsa-miR-122-5p	UGGAGUGUGACAAUGGUGUUUG
hsa-miR-20a-3p	ACUGCAUUAUGAGCACUUAAAG
hsa-miR-423-5p	UGAGGGGCAGAGAGCGAGACUUU
hsa-miR-629-5p	UGGGUUUACGUUGGGAGAACU
hsa-miR-101-3p	UACAGUACUGUGAUAACUGAA
hsa-miR-200c-3p	UAAUACUGCCGGGUAAUGAUGGA
hsa-miR-365a-3p	UAAUGCCCCUAAAAAUCCUUAU
hsa-miR-501-3p	AAUGCACCCGGGCAAGGAUUCU
hsa-miR-23a-3p	AUCACAUUGCCAGGGAUUUCC
hsa-miR-423-3p	AGCUCGGUCUGAGGCCCCUCAGU
hsa-miR-215	AUGACCUAUGAAUUGACAGAC
hsa-miR-376a-3p	AUCAUAGAGGAAAAUCCACGU
hsa-miR-320a	AAAAGCUGGGUUGAGAGGGCGA
hsa-miR-22-5p	AGUUCUUCAGUGGCAAGCUUUA
hsa-miR-338-3p	UCCAGCAUCAGUGAUUUUGUUG
hsa-miR-2110	UUGGGGAAACGGCCGCUGAGUG
hsa-miR-223-3p	UGUCAGUUUGUCAAAUACCCCA
hsa-miR-361-3p	UCCCCCAGGUGUGAUUCUGAUUU
hsa-miR-103a-3p	AGCAGCAUUGUACAGGGCUAUGA
hsa-miR-93-3p	ACUGCUGAGCUAGCACUUCCCG
hsa-miR-331-3p	GCCCCUGGGCCUAUCCUAGAA
hsa-miR-144-5p	GGAUAUCAUCAUAUACUGUAAG
hsa-miR-142-3p	UGUAGUGUUUCCUACUUUAUGGA
hsa-miR-210	CUGUGCGUGUGACAGCGGCUGA
hsa-let-7d-3p	CUAUACGACCUGCUGCCUUUCU
hsa-miR-199a-5p	CCCAGUGUUCAGACUACCUGUUC
hsa-miR-605	UAAAUCCCAUGGUGCCUUCUCCU
hsa-miR-766-3p	ACUCCAGCCCCACAGCCUCAGC
hsa-miR-19a-3p	UGUGCAAAUCUAUGCAAAACUGA
hsa-miR-584-5p	UUAUGGUUUGCCUGGGACUGAG
hsa-miR-144-3p	UACAGUAUAGAUGAUGUACU
hsa-miR-92a-3p	UAUUGCACUUGUCCCGGCCUGU
hsa-miR-126-3p	UCGUACCGUGAGUAAUAAUGCG
hsa-miR-363-3p	AAUUGCACGGUAUCCAUCUGUA
hsa-miR-148a-3p	UCAGUGCACUACAGAACUUUGU
hsa-miR-374a-5p	UUAUAAUACAACCUGAUAAGUG
hsa-miR-10b-5p	UACCCUGUAGAACCGAAUUUGUG
hsa-miR-190a	UGAUAUGUUUGAUAUAUUAGGU
hsa-miR-195-5p	UAGCAGCACAGAAAUAUUGGC
hsa-miR-29a-5p	ACUGAUUUCUUUUGGUGUUCAG
hsa-miR-125b-5p	UCCCUGAGACCCUAACUUGUGA
hsa-miR-18a-3p	ACUGCCCUAAGUGCUCCUUCUGG
hsa-miR-192-5p	CUGACCUAUGAAUUGACAGCC
hsa-miR-151a-3p	CUAGACUGAAGCUCCUUGAGG
hsa-miR-331-3p	GCCCCUGGGCCUAUCCUAGAA
hsa-miR-144-5p	GGAUAUCAUCAUAUACUGUAAG
hsa-miR-142-3p	UGUAGUGUUUCCUACUUUAUGGA
hsa-miR-210	CUGUGCGUGUGACAGCGGCUGA
hsa-let-7d-3p	CUAUACGACCUGCUGCCUUUCU
hsa-miR-199a-5p	CCCAGUGUUCAGACUACCUGUUC
hsa-miR-605	UAAAUCCCAUGGUGCCUUCUCCU
hsa-miR-766-3p	ACUCCAGCCCCACAGCCUCAGC
hsa-miR-19a-3p	UGUGCAAAUCUAUGCAAAACUGA
hsa-miR-584-5p	UUAUGGUUUGCCUGGGACUGAG
hsa-miR-144-3p	UACAGUAUAGAUGAUGUACU
hsa-miR-92a-3p	UAUUGCACUUGUCCCGGCCUGU
hsa-miR-126-3p	UCGUACCGUGAGUAAUAAUGCG
hsa-miR-363-3p	AAUUGCACGGUAUCCAUCUGUA
hsa-miR-148a-3p	UCAGUGCACUACAGAACUUUGU
hsa-miR-374a-5p	UUAUAAUACAACCUGAUAAGUG
hsa-miR-10b-5p	UACCCUGUAGAACCGAAUUUGUG
hsa-miR-190a	UGAUAUGUUUGAUAUAUUAGGU
hsa-miR-195-5p	UAGCAGCACAGAAAUAUUGGC
hsa-miR-29a-5p	ACUGAUUUCUUUUGGUGUUCAG
hsa-miR-125b-5p	UCCCUGAGACCCUAACUUGUGA
hsa-miR-18a-3p	ACUGCCCUAAGUGCUCCUUCUGG
hsa-miR-192-5p	CUGACCUAUGAAUUGACAGCC
hsa-miR-151a-3p	CUAGACUGAAGCUCCUUGAGG
hsa-miR-18b-5p	UAAGGUGCAUCUAGUGCAGUUAG
hsa-miR-28-3p	CACUAGAUUGUGAGCUCCUGGA
hsa-miR-335-5p	UCAAGAGCAAUAACGAAAAAUGU
hsa-miR-324-3p	ACUGCCCCAGGUGCUGCUGG
hsa-miR-204-5p	UUCCCUUUGUCAUCCUAUGCCU
hsa-miR-182-5p	UUUGGCAAUGGUAGAACUCACACU
hsa-let-7g-5p	UGAGGUAGUAGUUUGUACAGUU
hsa-miR-15b-5p	UAGCAGCACAUCAUGGUUUACA
hsa-miR-22-3p	AAGCUGCCAGUUGAAGAACUGU
hsa-miR-106b-3p	CCGCACUGUGGGUACUUGCUGC
hsa-miR-199a-3p	ACAGUAGUCUGCACAUUGGUUA
hsa-miR-29c-3p	UAGCACCAUUUGAAAUCGGUUA
hsa-miR-19b-3p	UGUGCAAAUCCAUGCAAAACUGA
hsa-miR-95	UUCAACGGGUAUUUAUUGAGCA
hsa-miR-29a-3p	UAGCACCAUCUGAAAUCGGUUA
hsa-miR-21-5p	UAGCUUAUCAGACUGAUGUUGA
hsa-miR-150-5p	UCUCCCAACCCUUGUACCAGUG
hsa-miR-30e-3p	CUUUCAGUCGGAUGUUUACAGC
hsa-miR-30b-5p	UGUAAACAUCCUACACUCAGCU
hsa-miR-543	AAACAUUCGCGGUGCACUUCUU
hsa-miR-24-3p	UGGCUCAGUUCAGCAGGAACAG
hsa-miR-29b-2-5p	CUGGUUUCACAUGGUGGCUUAG
hsa-miR-495-3p	AAACAAACAUGGUGCACUUCUU
hsa-miR-18b-5p	UAAGGUGCAUCUAGUGCAGUUAG
hsa-miR-28-3p	CACUAGAUUGUGAGCUCCUGGA
hsa-miR-335-5p	UCAAGAGCAAUAACGAAAAAUGU
hsa-miR-324-3p	ACUGCCCCAGGUGCUGCUGG
hsa-miR-204-5p	UUCCCUUUGUCAUCCUAUGCCU
hsa-miR-182-5p	UUUGGCAAUGGUAGAACUCACACU
hsa-let-7g-5p	UGAGGUAGUAGUUUGUACAGUU
hsa-miR-15b-5p	UAGCAGCACAUCAUGGUUUACA
hsa-miR-22-3p	AAGCUGCCAGUUGAAGAACUGU
hsa-miR-106b-3p	CCGCACUGUGGGUACUUGCUGC
hsa-miR-199a-3p	ACAGUAGUCUGCACAUUGGUUA
hsa-miR-29c-3p	UAGCACCAUUUGAAAUCGGUUA
hsa-miR-19b-3p	UGUGCAAAUCCAUGCAAAACUGA
hsa-miR-95	UUCAACGGGUAUUUAUUGAGCA
hsa-miR-29a-3p	UAGCACCAUCUGAAAUCGGUUA
hsa-miR-21-5p	UAGCUUAUCAGACUGAUGUUGA
hsa-miR-150-5p	UCUCCCAACCCUUGUACCAGUG
hsa-miR-30e-3p	CUUUCAGUCGGAUGUUUACAGC
hsa-miR-30b-5p	UGUAAACAUCCUACACUCAGCU
hsa-miR-543	AAACAUUCGCGGUGCACUUCUU
hsa-miR-24-3p	UGGCUCAGUUCAGCAGGAACAG
hsa-miR-29b-2-5p	CUGGUUUCACAUGGUGGCUUAG
hsa-miR-495-3p	AAACAAACAUGGUGCACUUCUU

**Table S3:** Ten significant upregulated miRNAs in patients with EH and T2DM.

miRNA ID	ED/N	*p*-value
hsa-miR-409-3p	2.391	0.327
hsa-miR-200c-3p	2.231	0.236
hsa-miR-33a-5p	2.153	0.161
hsa-miR-605	2.082	0.258
hsa-miR-543	1.986	0.318
hsa-miR-485-3p	1.912	0.215
hsa-miR-1	1.902	0.299
hsa-miR-497-5p	1.895	0.137
hsa-miR-10a-5p	1.884	0.031
hsa-miR-29b-2-5p	1.867	0.436

miR, microRNA; EH, essential hypertension; T2DM, type 2 diabetes mellitus; ED, essential hypertension with type 2 diabetes mellitus; N, healthy controls.

**Table S4:** Ten significant downregulated miRNAs in patients with EH and T2DM.

miRNA ID	ED/N	*p*-value
hsa-miR-195-5p	0.319	0.003
hsa-miR-130a-5p	0.353	0.001
hsa-miR-197-5p	0.372	0.309
hsa-miR-27a-5p	0.383	0.004
hsa-miR-374a-5p	0.389	0.031
hsa-miR-204-5p	0.404	0.108
hsa-miR-551b-3p	0.405	0.112
hsa-miR-99a-5p	0.416	0.301
hsa-miR-20b-5p	0.442	0.011
hsa-miR-885-5p	0.454	0.243

miR, microRNA; EH, essential hypertension; T2DM, type 2 diabetes mellitus; ED, essential hypertension with type 2 diabetes mellitus; N, healthy controls.

**Table S5 t05:** SNP in the miR-195-5p-binding region of DRD1 3′-UTR.

Base change	Position	Allele frequency	
		Control (*n=*100)	ED (*n*=100)
T/A	231	0.93/0.07	0.61/0.39
C/G	233	0.91/0.09	0.46/0.54

SNPs, single nucleotide polymorphisms; 3′-UTR, 3′ untranslated region; miR, microRNA; ED, essential hypertension with type 2 diabetes mellitus.

**Table S6:** Relative miRNA expression levels.

Name	N (healthy donor group)					E (essential hypertension group)					D (type 2 diabetes mellitus group)					ED (E with D group)				
	N-1	N-2	N-3	N-4	N-5	E-1	E-2	E-3	E-4	E-5	D-1	D-2	D-3	D-4	D-5	ED-1	ED-2	ED-3	ED-4	ED-5
hsa-miR-652-3p	27.74994	27.5214	26.23896	27.14213	24.93589	24.32263	26.86717	25.86409	26.73846	27.80176	27.12081	27.30996	28.1683	26.22699	27.48216	26.8101	27.64371	28.03684	26.98334	25.27692
hsa-miR-502-3p	30.2259	30.10943	29.7773	30.27932	28.40177	30.21685	30.85675	30.01515	29.86859	31.08951	30.62679	30.40215	29.83363	30.81268	30.43764	29.50065	30.59434	30.37207	29.79478	28.5667
hsa-miR-181a-5p	29.54401	29.57566	27.94752	28.77795	27.38079	25.18815	28.21215	27.12831	28.61724	29.11461	28.17641	28.47416	30.57065	27.47061	28.01435	29.15709	29.64834	29.88802	27.78866	27.23969
hsa-miR-339-3p	31.36388	32.40051	30.55619	31.90127	30.49482	28.37407	31.48838	30.51381	30.85313	31.85437	30.27144	31.43263	31.95248	30.29258	31.25646	31.55722	31.85877	31.3485	31.84089	29.59727
hsa-miR-221-3p	26.5359	28.22711	25.10158	25.71106	25.07761	21.84271	24.72138	24.28112	26.37955	26.3767	25.53376	26.59865	27.59387	24.44748	27.46118	25.97825	26.8859	26.76488	25.83128	24.66771
hsa-miR-409-3p	31.96837	38.06064	33.62046	32.75995	32.62264	27.68179	32.03365	31.9195	32.74971	35.14991	30.86067	34.08239	35.37194	29.96329	30.93717	33.50995	33.67864	31.52285	32.90079	30.58131
hsa-let-7f-5p	29.54303	29.93954	28.70305	29.26361	27.42158	25.53563	27.97526	27.91023	29.57095	29.82001	28.51415	29.4214	30.92477	27.65901	29.85581	29.6569	30.46705	29.90785	29.76985	29.69881
hsa-miR-154-5p	34.32638	36.63269	35.10727	35.29539	34.30911	37.98412	37.05836	36.92326	33.88621	36.93676	33.4407	32.09384	35.31864	32.00723	31.52567	35.91077	35.04369	35.08587	35.91708	32.61428
hsa-miR-27b-3p	28.00136	29.86563	26.67595	27.87492	25.19875	23.6541	26.27471	26.41524	27.72376	28.4739	27.2078	27.61225	28.96364	25.70977	28.93992	27.64356	28.91744	28.32699	28.06383	26.05521
hsa-miR-155-5p	33.69112	33.86313	31.35646	32.51689	31.94231	29.92997	32.84324	31.56445	31.83759	33.48145	32.18735	32.78399	34.12407	31.67273	31.68852	33.5486	35.02567	33.00285	33.54777	33.24107
hsa-miR-374b-5p	30.44037	30.39161	28.71487	29.30584	27.41831	26.5144	28.78996	28.74502	29.58283	30.49701	28.70949	30.28804	31.29231	27.99418	29.6185	29.90086	30.93332	30.5164	29.81221	28.0003
hsa-miR-140-3p	26.59032	26.13342	25.87901	26.76519	24.22189	26.73265	27.04536	25.98797	26.03578	27.16548	26.44001	26.21607	26.11365	26.98335	26.79785	25.30769	26.18356	26.60085	26.50047	25.08133
hsa-miR-93-5p	24.46973	23.68834	23.50438	24.14214	21.15665	22.87703	25.23616	23.3616	22.91347	25.20253	23.86945	23.80836	24.2531	24.35304	24.11983	23.57152	24.46046	24.82886	23.94189	22.47515
hsa-miR-92b-3p	33.44775	34.48301	33.60299	33.9209	33.747	33.03883	33.51449	34.24391	34.80063	34.24825	33.98491	34.25697	34.2988	33.27515	35.95933	33.64039	35.21371	35.0135	32.81757	32.58928
hsa-miR-200a-3p	35.81265	38.98334	33.88347	34.43107	33.55525	33.82429	34.22566	35.04956	34.51918	38.24019	35.71024	33.16651	35.51819	35.63606	33.54199	33.23368	34.69623	35.67212	34.91472	34.75449
hsa-miR-505-3p	31.6744	31.90579	30.44626	31.41989	28.23391	29.68319	30.87034	30.72041	32.1862	31.49747	30.64051	29.96584	31.90407	30.08533	30.9188	31.36671	32.1033	31.57569	32.28345	30.08773
hsa-miR-23b-3p	27.61481	29.69939	26.08749	26.75518	24.41731	23.32742	26.15857	25.65728	27.48725	27.53347	26.50705	27.11682	28.67356	25.53107	27.39611	27.20536	27.6649	27.88485	26.9346	25.24746
hsa-miR-484	26.49639	25.92376	25.3414	26.28128	24.53492	24.33617	26.44572	24.85307	25.60827	26.54247	25.83553	25.49558	25.75777	25.99555	25.99618	25.21359	25.95281	26.35073	25.89331	24.86213
hsa-miR-141-3p	35.24291	34.65108	32.97037	33.28511	31.5345	31.35641	32.457	33.04189	34.0213	37.80652	34.66799	35.03629	34.25009	33.83165	32.58475	33.08614	33.38719	32.91007	34.46541	33.08076
UniSp3 IPC	20.83079	21.03874	20.97121	20.86166	21.05546	21.12578	21.0701	21.48895	21.81917	21.03903	20.92505	20.89359	20.90363	20.89149	21.67327	20.9502	21.01185	21.01761	20.91675	21.25114
hsa-miR-10a-5p	37.82522	38.1038	35.64824	37.69761	35.18068	33.68993	35.55281	35.26032	37.86531	36.75699	34.3638	36.46663	37.19475	36.22292	36.27803	35.83612	36.47287	36.37754	35.891	34.71648
hsa-miR-106a-5p	23.88025	23.17466	22.8301	23.46748	21.1071	21.88073	24.02368	22.61029	22.34813	24.51563	23.35664	23.31617	23.92934	23.87537	23.79013	23.34193	24.20332	24.36314	23.68401	21.9606
hsa-miR-27a-3p	31.58713	32.98496	30.04809	30.9465	28.51177	27.05272	29.31883	32.19897	32.12651	31.7395	30.82221	30.96539	32.22275	28.26848	33.39303	31.07388	32.79878	31.52774	30.97642	29.82726
hsa-miR-145-5p	28.08021	30.09938	27.49263	27.98185	25.90704	26.24076	27.82468	28.6746	29.511	29.21755	27.45531	27.64322	28.39865	26.49436	29.06153	27.93881	29.10808	29.10705	28.70984	27.24894
hsa-miR-1	33.44626	38.73879	35.06868	36.06837	35.488	32.42819	34.57653	35.34016	34.9604	37.65683	36.06906	36.75673	38.1973	31.90985	36.37341	34.17597	34.81035	35.6044	34.57869	33.7767
hsa-miR-185-5p	26.74238	25.62303	26.13874	27.10768	24.71559	25.58529	27.46045	27.38226	26.56527	27.78893	26.52845	26.33804	26.70703	26.89922	27.79907	25.58553	26.66526	27.28644	26.23623	25.36239
hsa-miR-382-5p	33.20625	36.29013	34.48356	32.96202	35.24107	29.57438	32.53288	32.76921	33.75302	35.46727	33.21151	33.82644	32.32356	31.87567	31.01063	35.51333	34.95464	33.65596	32.94765	31.60677
hsa-miR-486-5p	21.8098	21.41052	21.78252	22.217	20.28876	22.90534	23.14312	21.79153	21.52337	22.69885	21.54243	21.33224	21.81741	22.88875	22.58685	20.79542	21.97221	22.33524	21.70398	21.22345
hsa-miR-32-5p	30.03758	29.06372	28.96416	29.30087	27.07852	28.30837	29.17505	28.73644	29.27338	29.78134	30.05366	29.15879	29.46026	29.71922	30.83629	28.40371	28.51788	29.66493	28.68057	27.59264
hsa-miR-26a-5p	27.37512	28.07665	25.75058	26.57973	24.09008	23.22924	25.82531	25.87128	26.90529	27.33678	26.14462	26.82854	28.22435	25.18349	27.47953	27.33305	28.31028	27.85763	26.9539	25.18696
hsa-miR-133b	31.78462	35.69064	32.35449	34.1036	32.78946	30.92826	33.01174	31.86402	33.24655	33.6403	33.99249	33.56842	33.57713	29.91051	32.3575	31.7153	32.10364	34.17896	32.23587	31.50515
hsa-miR-143-3p	30.68927	32.58359	30.24534	31.91434	27.04269	29.36839	31.41239	30.34436	31.00669	32.02716	30.76045	31.57299	30.97994	29.58453	30.28759	31.27313	30.58678	31.81768	30.40353	29.31771
hsa-let-7d-5p	29.06523	28.92637	27.73608	28.1984	26.71953	24.7453	27.53663	27.4304	28.91053	29.06821	27.8516	28.77179	29.93562	27.04076	29.24649	28.34693	29.86373	29.17829	28.4986	26.76672
hsa-miR-30a-5p	32.54282	33.56037	31.08787	31.929	29.76558	30.63907	31.43457	30.14122	32.97329	32.44356	31.27983	30.61799	31.8724	31.7149	33.75945	31.44139	32.69341	31.82043	31.44052	30.96382
hsa-miR-133a	31.7527	36.39534	31.89617	33.0822	31.62113	30.89086	32.51809	32.61858	33.02648	32.43336	33.38371	32.45379	33.2342	29.35147	32.9175	31.50357	32.04395	33.06936	32.41431	31.14165
hsa-miR-222-3p	27.68221	27.50594	26.40857	27.68184	24.89188	25.32533	27.14656	25.12012	26.07643	27.65367	27.05235	26.88651	27.5361	26.86411	26.77897	26.69998	27.5412	27.27179	27.32041	26.14155
hsa-miR-20b-5p	31.66225	30.92777	31.70095	31.58779	29.40001	32.7573	32.53058	31.27701	30.40097	32.25677	31.60289	31.78093	31.64437	32.26431	32.50222	31.55463	32.50263	33.67727	32.75087	30.18692
hsa-miR-342-3p	29.89237	29.63649	26.8432	28.16519	26.61326	26.41042	29.11737	26.63868	27.28963	29.24671	26.34892	27.30435	28.63808	26.38236	26.91957	28.05408	28.63852	29.14133	28.36472	27.23791
UniSp3 IPC	20.12914	20.28845	20.25332	20.26003	20.20704	20.44624	20.62674	20.66848	20.94368	20.34843	20.3503	20.40245	20.26865	20.23343	20.82561	20.32219	20.29822	20.49263	20.19533	20.57718
hsa-miR-328	30.53617	31.93652	29.36688	30.37124	29.14912	27.0294	29.92333	28.56399	30.14203	32.02518	30.14473	30.4854	31.83453	29.59551	30.59017	30.17805	30.57367	31.58248	30.78035	28.99282
hsa-miR-324-5p	31.54158	30.34379	29.76351	30.50898	28.56602	27.89933	30.71882	29.8955	29.66767	31.92703	29.93047	31.16385	31.79148	29.73488	30.60059	30.20649	31.07916	31.87018	31.50987	28.61568
hsa-miR-532-3p	30.91136	30.6021	29.60564	30.13448	28.46165	29.17072	31.22001	29.06131	30.318	31.14449	30.18206	29.8714	30.09981	30.43989	30.44165	29.77946	30.44059	30.64341	30.70388	29.36934
UniSp3 IPC	21.0251	21.1843	21.0683	21.02428	20.86283	21.27783	21.2916	21.61786	21.82626	20.9291	21.15723	21.0395	21.05303	20.89177	21.81205	20.95441	21.12836	21.18615	20.96256	21.22254
hsa-miR-15a-5p	25.1894	24.59376	24.23149	24.98174	22.69894	24.10404	24.88989	24.65292	24.35845	25.23549	25.27914	24.78283	24.33446	25.27877	26.16628	23.69828	24.94342	24.75539	24.79979	23.30074
hsa-let-7e-5p	30.35847	32.25116	29.30433	29.61547	28.48113	25.80256	28.39368	29.44182	30.68124	30.53901	29.31629	30.52857	30.8044	28.96209	29.85656	29.73802	31.08312	31.19833	30.18432	29.34503
hsa-miR-532-5p	30.70271	30.20063	29.86886	30.05227	28.0432	29.54509	30.75792	29.9071	30.25387	30.56456	30.33393	30.11314	30.50165	30.68427	30.90021	29.70996	30.64305	30.41774	30.09644	28.85405
hsa-miR-139-5p	30.95367	32.44723	28.65154	29.53386	29.18265	27.16527	29.13087	28.40357	30.41887	30.6824	29.50002	30.37273	31.34886	28.45693	31.17527	30.64484	31.9709	30.05839	30.3386	28.69988
hsa-miR-194-5p	31.02555	29.47414	29.51723	30.14846	24.50118	30.29932	30.30292	29.93296	29.48233	30.43364	29.30925	28.66019	30.25448	30.45884	30.09222	29.63083	30.49415	30.20627	30.51679	28.80742
hsa-miR-375	32.59305	34.16055	32.32737	32.53798	29.59822	32.27096	31.01291	31.55889	35.12284	32.78225	30.71932	29.54231	31.60568	32.19861	34.78196	29.98617	30.91087	33.37708	33.07339	33.10255
hsa-miR-660-5p	28.21718	27.24476	26.78669	28.15467	25.0884	27.9019	27.86754	26.45333	26.72004	28.1124	27.85169	27.53834	27.36163	28.22911	27.51835	26.94085	27.83518	27.5827	27.68299	26.18647
hsa-miR-451a	18.48677	17.46424	17.48924	18.30064	15.18831	19.38609	18.59371	17.71829	17.13805	18.65624	17.92601	17.51848	17.51177	18.96806	18.7079	17.20353	18.13001	18.42848	18.02586	16.67711
hsa-miR-574-3p	30.30313	32.10363	29.35911	30.5051	27.7014	27.84048	29.86473	28.73419	30.71109	30.9359	29.85131	29.04526	31.34966	29.35229	30.58157	30.15318	30.58788	31.8007	30.62631	29.70512
hsa-let-7a-5p	25.83652	25.76985	24.54204	24.96715	23.49537	22.12276	24.56216	23.9065	25.5391	25.50868	24.68851	25.44837	26.52234	24.11973	25.39686	25.30265	26.06368	25.82883	25.32109	23.35952
hsa-miR-551b-3p	34.82532	35.38679	33.09311	34.55676	31.66249	29.36407	32.92147	31.11393	33.36134	30.4085	34.31084	33.78183	33.93639	32.27083	34.45362	33.64428	33.83847	35.76249	36.14222	36.16309
hsa-miR-128	28.83977	28.75929	28.05537	28.3804	26.66791	26.35832	28.94085	27.55607	28.27582	29.61382	28.58958	28.47557	28.81084	28.43447	29.3721	27.84185	28.55545	29.25512	28.09641	26.98679
hsa-miR-130a-3p	29.41045	29.12493	28.49799	29.20877	26.51403	26.27691	28.55954	27.63877	28.80502	27.51954	29.7015	29.23774	29.61988	28.65895	31.52462	28.50282	28.64103	29.78223	27.90754	26.8956
hsa-miR-125a-5p	29.5871	32.28732	29.0844	28.54903	27.80309	25.65482	28.55138	28.81979	30.28619	29.83327	29.29193	29.48373	31.12437	28.97035	28.83739	29.04146	30.14285	30.51418	29.57005	28.46987
hsa-miR-28-5p	31.96777	33.49418	29.86786	31.09059	29.33175	30.81703	30.2412	29.72644	31.34855	31.63658	30.87022	31.75866	34.60873	29.79343	30.81774	32.03813	33.71215	32.33774	31.80047	29.1291
hsa-miR-485-3p	36.83944	36.15921	34.96649	32.98107	34.30843	29.79036	34.21365	33.45903	35.47761	36.79858	33.22994	36.1912	35.80698	31.94852	33.53072	34.01696	34.85069	34.27777	34.66191	32.28302
hsa-miR-497-5p	33.79824	35.26284	31.51067	34.26224	31.63743	31.15742	32.94233	31.74152	35.81569	33.64935	32.99963	31.92582	32.22111	32.61117	34.64199	32.18248	32.53742	32.81676	33.24314	30.59141
hsa-let-7b-3p	31.96007	33.13511	31.27911	30.86678	30.59552	30.5542	31.48372	29.57355	33.34782	33.48306	31.9468	30.50366	31.9083	31.72993	31.6154	30.76108	31.2113	31.40703	31.21127	30.38604
hsa-miR-425-3p	31.23219	31.62689	30.10234	31.40576	28.13178	27.86525	31.29121	31.0194	31.09642	32.55162	30.39668	31.30321	32.08244	29.71292	31.52193	30.46222	31.99404	31.80791	30.75182	29.72478
hsa-miR-132-3p	31.86506	32.28435	31.21421	31.55189	28.77818	29.83697	31.49297	29.85232	31.03606	31.87405	31.33688	31.22271	30.97756	31.1991	30.75614	30.23074	31.73812	31.51785	30.9658	30.46886
hsa-miR-25-3p	23.75698	22.97704	23.20976	23.96692	21.38269	23.6522	24.84752	23.01605	22.41704	24.52008	23.70584	23.45254	23.71441	24.33735	23.36906	22.60993	23.76549	23.95233	23.68305	22.08658
hsa-let-7c	31.46542	31.38333	30.07255	30.23474	28.09053	27.28307	29.82328	30.31844	31.88651	31.42961	30.07534	30.49305	32.35527	29.02953	32.19058	30.83719	32.37699	31.34012	30.39614	29.62025
UniSp3 IPC	21.06835	21.14273	21.1418	20.90977	21.33057	21.23945	21.29383	21.45312	21.79418	20.99621	21.19244	21.06163	21.10684	21.06488	21.89692	21.11966	21.21782	21.2475	21.01051	21.26308
hsa-miR-18a-5p	28.97923	27.89222	27.63545	28.0697	25.98354	25.69059	28.33493	26.8864	27.09946	28.60085	27.78891	28.31283	28.84043	28.44304	28.03572	28.04255	28.77613	29.06246	27.78856	26.34025
hsa-miR-33a-5p	32.97207	36.15842	31.90488	32.62524	31.75563	28.8476	31.71632	30.68616	33.47268	33.15192	33.268	32.73966	33.05554	31.2481	35.57744	32.0154	32.57646	32.57681	31.88531	30.34244
hsa-miR-29b-3p	30.20344	29.24642	29.19008	29.84276	27.14238	28.68591	29.76318	29.53538	28.57231	30.54786	29.55242	29.07911	29.94887	29.13524	30.25496	28.90917	29.73547	30.34319	29.52467	27.66038
hsa-miR-16-5p	20.1034	19.65565	19.08557	19.97363	17.79215	20.00053	20.33983	18.8456	18.72069	20.58713	19.92686	19.68035	19.40052	20.81589	19.73464	18.9951	19.633	19.90282	19.83699	18.36417
hsa-miR-136-5p	36.34523	39.34459	35.4565	35.38076	34.4506	29.69584	36.30419	33.92336	34.35291	35.63195	32.9208	34.28623	39.74125	31.41521	32.79159	33.35181	34.01268	34.19381	34.33761	30.15464
hsa-let-7b-5p	25.25075	24.60535	24.59361	24.91743	22.61386	24.04738	25.98504	25.20323	25.04149	26.07109	24.87489	24.76023	25.83438	25.34926	25.61287	24.89552	25.77196	26.39036	24.9645	23.96882
hsa-let-7i-3p	33.99617	34.63456	33.09503	36.62936	31.78859	31.46923	33.33028	32.4646	35.60875	33.30779	33.41705	34.45986	34.53857	32.9489	36.07883	33.14796	34.50572	34.83747	33.59931	32.26686
hsa-miR-152	30.22637	30.69527	29.26405	30.62641	27.58311	27.17961	29.78639	28.64776	30.2661	31.12018	29.73712	29.95367	30.33964	29.21488	30.46405	29.73417	30.98352	30.53451	30.52707	29.08578
hsa-miR-106b-5p	26.21872	25.4469	25.4626	25.73227	23.33224	24.99771	26.6251	26.26454	26.54981	26.7477	25.99509	25.53234	26.3705	26.50316	27.26551	25.58824	26.77437	26.62033	25.74315	25.05995
hsa-miR-30c-5p	28.37185	28.64766	26.76522	27.31799	25.45133	24.66385	26.98539	26.10352	28.28804	28.33102	26.78388	27.60242	28.70506	26.25275	27.88244	27.27392	27.80614	27.83106	27.39672	25.74457
hsa-miR-15b-3p	27.83004	26.95826	27.13908	27.51223	24.99949	26.54021	27.89947	26.49076	26.03033	27.69174	27.63359	27.46765	26.98485	27.8718	27.43242	26.2113	26.87783	27.5419	27.15345	25.63427
hsa-miR-197-3p	30.33917	32.13727	28.78687	29.41822	27.26965	27.06521	29.50554	28.41073	30.59773	30.4379	29.29697	29.51136	31.8563	28.34583	29.34933	29.21643	29.82783	30.19058	29.60906	28.00565
hsa-miR-142-5p	29.96804	30.20316	28.15688	29.1513	26.17647	26.65147	28.54387	28.14291	29.29087	29.89351	29.03771	29.51081	30.37655	28.00583	29.95366	28.78869	29.22298	29.82101	28.56376	27.30625
hsa-miR-99b-5p	30.7004	32.89823	30.56781	30.1983	29.4475	26.78618	30.05549	29.86919	32.12754	31.33938	30.5717	31.09753	31.50773	30.33964	30.55146	30.24098	30.97712	31.96217	30.83422	30.1991
hsa-miR-296-5p	32.10905	30.91676	31.03687	31.72988	30.3647	33.78673	34.98366	30.78675	32.3936	33.70561	32.91222	31.30762	31.65395	32.18618	32.62407	31.28663	31.11049	32.37693	31.37152	29.50946
hsa-miR-30e-5p	26.61948	26.57397	25.899	26.79168	23.89947	24.92905	26.77394	26.01474	26.1682	26.9855	26.7092	26.396	26.53903	26.51903	27.27281	25.74514	26.62144	26.72083	26.75272	25.33763
hsa-miR-326	32.81226	34.08975	30.67696	31.02576	29.99435	27.49887	31.10153	30.67121	30.85221	32.48916	30.84852	31.9943	34.36186	30.20116	32.96125	32.40442	31.69891	33.60544	31.06181	29.38402
hsa-miR-146a-5p	28.85095	30.44415	26.69238	28.24585	26.97342	24.42608	27.65545	26.59086	28.15089	28.85262	26.75819	28.46425	29.09837	26.50301	28.96867	27.87789	28.8551	28.99847	27.61624	26.53414
UniSp3 IPC	20.38931	20.55089	20.50438	20.47939	20.41287	20.42801	20.76364	20.62329	21.03287	20.52586	20.56365	20.57537	20.45766	20.55502	21.0418	20.56197	20.44547	20.56457	20.37643	20.61126
hsa-miR-421	34.28639	33.37249	32.75791	32.75096	30.73877	30.26873	32.85915	31.63228	32.05	33.19094	32.31892	32.78222	33.62451	31.94183	33.04474	32.08919	33.46824	33.80203	33.1664	31.27593
hsa-miR-424-5p	31.76299	32.27545	30.62981	32.28143	27.88008	30.86078	31.74688	30.94585	32.34484	32.25546	31.8635	31.219	31.4844	31.41708	32.66533	31.23167	32.18581	31.91502	30.87414	30.09237
hsa-miR-223-5p	35.32374	34.57774	32.54866	33.78231	29.17605	31.431	33.64744	31.06877	32.93565	35.26397	33.99865	33.27614	34.06653	32.02735	31.70437	32.66264	34.20536	33.90226	33.72893	32.94328
hsa-miR-146b-5p	33.0107	33.87771	31.1506	32.37305	29.9455	29.67134	32.23698	32.94191	32.63085	33.10917	30.22475	32.92095	33.21209	30.42843	32.67757	33.01573	34.34956	34.03154	32.02308	31.73014
hsa-miR-107	29.08695	28.74306	27.24541	28.19932	25.43937	25.09633	27.76247	28.23588	29.725	28.43518	28.02919	28.30266	29.45064	27.03219	31.06113	28.16983	29.38462	29.13248	28.10419	27.29096
hsa-miR-205-5p	34.7469	34.37409	34.29313	33.98984	32.98815	34.0923	33.43979	32.87469	39.40604	33.72156	34.36265	32.58087	33.58212	34.79503	37.6055	32.76176	34.69407	35.40566	32.14544	32.68945
hsa-miR-148b-3p	27.66843	27.3128	26.59612	27.73573	24.60881	24.83809	27.61614	25.7985	25.96808	28.16562	27.32233	27.4031	27.63031	26.89429	27.40427	26.6445	27.49121	27.81897	27.24875	25.39712
hsa-miR-339-5p	32.09259	33.79491	29.99133	31.09841	30.92351	27.53405	30.52344	29.97265	31.91004	32.08718	30.61545	31.40527	33.11576	29.74201	31.8802	31.8997	32.3461	31.86757	31.74854	29.57191
hsa-miR-20a-5p	23.63481	22.95393	22.79511	23.21	21.07748	22.22794	23.96483	22.8081	22.38545	24.31785	23.47965	22.96101	23.63756	23.88515	23.82668	22.81731	23.99888	24.0398	23.96101	22.02838
hsa-miR-17-5p	30.44374	30.12178	29.82937	30.58703	27.80768	28.49124	30.77487	29.61528	29.70941	31.20881	30.3924	29.99724	30.53359	30.37871	31.89964	29.72308	31.61963	31.74181	30.64851	29.13463
hsa-miR-500a-5p	31.999	30.67757	30.88746	31.49914	29.23199	31.6266	33.08257	32.71986	32.19905	33.15011	31.63047	32.10685	34.39571	32.72407	32.00803	32.27414	32.68087	33.42032	32.25312	30.67178
hsa-miR-30d-5p	31.03676	31.03976	29.43331	30.35775	28.53174	28.02511	30.7582	30.16576	31.38217	31.54103	30.01038	30.52382	30.82912	29.72977	31.51934	30.15021	30.50127	31.09861	30.12171	29.55901
hsa-miR-378a-3p	29.07881	28.89016	28.21669	29.24694	26.09562	28.50094	29.65974	27.90733	28.29309	30.21653	28.9845	27.958	28.6598	28.66897	28.90623	28.44772	29.20752	29.41204	28.83443	28.09078
hsa-miR-186-5p	28.21332	27.62693	27.5058	28.20357	25.33418	26.22033	28.52195	27.08626	26.83163	28.70276	27.38388	27.83187	28.04749	27.34616	27.33942	26.97155	27.9403	28.42387	28.46801	26.25981
hsa-miR-425-5p	25.98755	25.59435	25.24202	25.83141	23.63913	25.03722	26.57786	25.13631	24.97655	26.44872	25.63775	25.41631	25.76954	26.08943	25.77297	25.20617	25.98444	26.03029	26.34618	24.23706
hsa-let-7i-5p	27.35768	26.86716	26.38713	26.99316	25.01061	24.64456	27.10329	26.92386	26.5123	28.05332	27.00024	27.49013	27.70028	26.17175	28.51793	26.46903	27.9125	27.71668	27.7384	25.89654
hsa-miR-346	32.97495	35.94007	32.91123	33.93928	33.70149	33.73249	34.42959	31.81371	35.00037	32.57941	33.29454	33.96266	34.68408	36.64476	33.94651	34.34383	34.75624	33.18891	32.75082	33.88271
hsa-miR-26b-5p	29.32872	28.88938	28.11414	28.69153	25.82461	26.31993	28.90173	28.05593	28.39873	26.93873	27.92162	28.08031	29.82573	28.01764	29.51037	29.24134	30.11016	29.79684	28.88724	27.32803
hsa-miR-193b-3p	31.56719	33.07303	31.28595	31.72584	27.5653	30.43094	32.66721	31.42588	32.07764	33.36612	29.32218	27.17879	31.51554	31.47475	32.66997	31.63136	32.2887	31.69425	28.71753	29.08636
hsa-miR-34a-5p	34.06146	34.57602	31.89062	33.01111	30.35692	30.62814	32.94653	30.25604	33.65652	33.69281	31.99438	30.33749	32.48767	32.2882	33.98626	32.14907	32.38044	32.59329	32.02737	31.32315
UniSp3 IPC	21.00049	21.11922	21.10762	20.8879	21.05193	21.21672	21.28537	21.6184	21.82974	21.0888	21.28841	20.9936	21.09703	21.11533	21.70503	21.11499	21.17326	21.20608	21.03429	21.2736
hsa-miR-320b	26.95977	26.87001	26.15631	27.15481	24.50027	25.43245	26.90346	25.60264	26.31562	27.59269	26.7624	26.41115	26.45954	26.70227	27.56089	26.12977	26.68162	27.00547	26.57443	25.86712
hsa-miR-885-5p	32.69805	34.5323	32.2734	32.13612	27.95339	32.89874	30.99129	30.95694	36.17082	33.80361	30.79626	27.96267	32.48107	31.04595	30.62359	31.75146	32.98113	33.11964	33.17522	33.77075
hsa-miR-590-5p	29.62653	29.52869	29.13459	29.516	26.54636	27.76521	29.66522	27.94672	28.42055	29.16378	29.55649	29.67328	29.19773	29.82452	29.12372	28.45526	29.07391	29.90583	29.84341	27.71594
hsa-miR-127-3p	34.42763	36.93448	37.8028	33.25026	32.91518	33.22889	34.65968	33.17507	33.67817	34.15639	32.76609	34.73983	33.2252	31.35259	31.00222	34.45742	37.37908	34.64361	35.42044	34.66831
hsa-miR-191-5p	27.59792	28.21453	26.0598	26.92878	24.49451	24.05631	26.86379	26.57628	27.53786	28.23863	26.63509	27.49481	28.13501	26.08828	28.24809	27.35439	28.44825	28.17356	27.11774	25.80836
hsa-miR-99a-5p	31.96146	33.06466	30.19206	31.46272	24.67068	29.64922	30.6739	30.32564	32.95655	31.29417	30.21591	29.07325	31.99554	31.12979	32.99957	30.84759	32.09533	31.29944	32.01439	30.9326
hsa-miR-16-2-3p	29.94031	30.1285	30.28909	30.17707	27.17864	30.52811	30.67544	31.19754	31.83175	30.63953	29.81005	29.2398	29.71399	30.30368	32.63086	29.07293	30.6186	30.26246	29.07335	29.70464
hsa-miR-301a-3p	29.45958	30.21708	28.82285	29.59778	27.17686	26.68168	29.09125	28.04389	29.33576	30.19157	29.76094	29.47846	30.25572	29.43878	30.45325	30.0342	30.30558	30.86009	29.7785	27.98571
hsa-miR-140-5p	30.0195	30.21419	28.93958	30.18019	26.78533	27.18782	30.00977	28.51517	29.56227	30.45989	29.48979	29.7663	30.28615	28.46059	29.5461	28.85058	30.38588	30.46991	30.38224	28.42335
hsa-miR-151a-5p	27.17035	27.18094	25.86517	26.77729	25.14858	23.28316	25.94074	25.71939	27.03396	27.39928	26.32515	27.16764	27.84965	25.42245	27.74338	26.90835	28.02356	28.01242	27.67413	25.71401
hsa-miR-130b-3p	32.49076	32.67072	31.471	32.37576	30.18452	29.82869	31.91692	32.12663	32.69338	33.37513	31.87758	32.4603	32.10622	31.66996	34.01106	31.39933	32.31535	32.40686	31.96604	30.91024
hsa-miR-122-5p	29.75502	29.78442	27.10293	28.70412	27.79973	26.71362	27.54651	27.40428	31.30482	29.43769	25.64243	23.41682	29.61247	27.4189	32.06776	28.45429	28.69315	29.56806	28.51405	29.51792
hsa-miR-20a-3p	35.50993	35.13137	32.36831	34.00836	34.36166	30.70102	33.55255	33.10722	32.23634	34.29068	33.38481	34.10626	34.85632	34.68712	34.7523	35.65752	34.55519	34.24951	35.33783	33.13094
hsa-miR-423-5p	26.64392	26.57219	25.78012	26.30782	24.83073	25.32058	27.01967	25.38796	25.46094	26.92381	26.43603	26.30937	26.48308	26.399	26.4995	25.31666	25.44091	26.61329	25.5438	24.73648
hsa-miR-629-5p	31.44825	31.57955	29.95617	33.78228	29.47496	30.71213	31.26098	32.63531	30.89545	32.56001	32.5333	31.2678	31.27802	31.66961	32.31483	30.02264	31.09699	32.21756	32.50428	29.87607
hsa-miR-101-3p	26.97421	25.40171	25.69379	26.37957	23.5324	24.87663	26.45146	25.30316	24.63843	26.72278	26.79647	25.98135	25.70853	26.92004	25.91345	25.40806	26.03297	26.34132	26.17399	24.6391
hsa-miR-200c-3p	34.8623	35.25546	33.21792	33.02517	32.7479	29.54656	33.14934	35.73673	33.75283	39.08882	32.84033	33.312	34.82242	32.1915	32.1393	33.69103	34.30943	33.33028	32.44981	32.19209
hsa-miR-365a-3p	32.67135	31.8509	30.73993	32.28486	27.3191	31.28847	32.62153	31.06773	31.54612	32.00368	31.04312	28.96717	30.93914	30.82579	30.70286	30.54227	31.21945	31.97037	29.07414	29.44951
hsa-miR-501-3p	33.77105	32.76588	31.79238	32.72643	31.66015	32.8028	33.24609	32.67595	32.4588	32.96821	33.91291	33.53212	32.47366	33.28887	33.35595	31.97056	32.92544	33.35352	32.56938	31.0937
hsa-miR-23a-3p	25.4937	27.17482	23.99858	25.04817	22.82148	21.65174	24.54852	23.74756	25.3092	25.56107	24.90077	25.20611	26.34066	23.61326	25.11126	25.08897	25.89022	25.91361	25.13085	23.46818
hsa-miR-423-3p	28.77637	28.97673	27.11412	27.66028	26.82952	24.90534	27.61923	26.91311	27.72387	28.70288	27.8058	27.87058	29.48853	26.38052	28.05927	27.82365	28.39541	28.98639	28.47561	26.15592
hsa-miR-215	29.44474	28.17284	28.5431	29.54204	24.5214	29.8439	29.61881	28.34815	28.29208	29.50648	28.38792	27.19236	29.31909	29.54315	29.10647	28.18778	29.15725	29.52944	29.46916	27.96056
hsa-miR-376a-3p	36.51092	39.01264	37.24707	35.98175	36.59857	31.8899	36.9487	37.93353	38.25774	37.89752	35.04425	37.42385	39.41479	34.83206	36.52796	38.81685	37.89062	37.81674	38.27707	35.32907
hsa-miR-320a	27.8443	27.48381	26.75114	28.01059	25.2477	26.17948	27.93927	27.73173	27.9886	28.41023	27.34566	27.52831	27.51764	27.07989	29.14034	26.90961	27.89774	27.84139	27.4643	26.94567
hsa-miR-22-5p	32.62365	32.34061	30.23085	32.1987	29.67163	29.16887	31.50285	30.91357	31.94666	32.54826	31.44608	31.20769	32.90216	30.55532	33.09369	30.7609	31.68508	32.39146	30.12033	30.21621
hsa-miR-338-3p	32.58246	32.50407	29.90822	32.3019	27.34408	28.10741	31.23338	29.76016	30.00632	31.44597	31.17906	31.67855	33.25192	29.1628	30.93922	30.75279	31.6276	31.61378	31.02343	29.27295
hsa-miR-2110	32.4483	32.1643	31.93545	32.0053	30.66987	30.71891	33.22841	30.92765	31.85031	32.51696	32.09001	32.5285	32.27613	32.54137	31.81155	31.19553	31.90546	33.44205	31.97486	30.94209
hsa-miR-223-3p	23.38143	24.41653	21.55545	22.68441	18.57904	19.74873	22.42085	21.36249	22.00883	23.00233	22.3176	22.77316	24.46379	21.05343	21.47469	22.72187	23.34807	23.55475	23.11802	21.02463
hsa-miR-361-3p	36.01968	35.69118	34.00154	34.39228	32.52074	32.03904	34.96273	35.28903	37.48752	36.02328	33.71529	35.12146	34.67405	32.95112	34.08602	34.33622	35.59987	35.95909	35.17646	34.96614
hsa-miR-103a-3p	26.93517	26.89095	25.66184	26.22617	24.55992	23.05624	25.88631	26.38374	26.87309	27.19262	26.44444	26.54104	28.15291	25.23928	28.57471	26.77884	28.23468	27.70967	26.45439	25.14162
hsa-miR-93-3p	29.8402	29.72053	29.00847	29.2478	27.57036	28.35633	30.50112	29.17769	28.99668	30.3149	29.37268	29.26221	30.2138	29.95811	29.63111	29.93006	29.92666	31.10624	30.05894	28.2526
hsa-miR-331-3p	31.33761	31.85614	30.39974	31.22844	29.41814	27.70556	30.7745	30.38876	31.70765	31.1916	30.7668	30.80864	32.14602	29.70831	31.76879	30.70878	31.92244	32.29941	31.28941	29.84973
hsa-miR-144-5p	30.5463	28.30142	28.60622	29.28318	27.17848	30.53761	30.42263	29.09061	29.87156	29.14756	28.94647	29.26965	29.81558	30.7608	29.37571	29.17263	29.94614	29.30577	28.89575	27.33498
hsa-miR-142-3p	26.91883	27.58633	25.21892	26.30003	24.71534	23.27428	24.90311	25.22482	27.20145	26.85488	25.84844	26.71925	27.82249	24.52527	27.36963	26.58507	27.85983	26.99565	27.36211	25.07437
hsa-miR-210	30.28277	29.97678	29.21919	30.14465	28.89626	29.28686	29.89688	28.71085	28.81251	30.61754	30.52575	29.44265	29.8384	30.38413	30.11612	29.27381	29.67077	30.35948	29.69189	28.25408
hsa-let-7d-3p	27.39229	28.91246	26.92497	27.34841	25.89912	24.91726	27.33879	26.2551	28.1826	28.46649	27.35308	27.08252	28.25494	26.57891	28.28829	26.9092	27.81939	28.09588	27.69543	26.4758
hsa-miR-199a-5p	30.74037	32.36673	28.48457	29.20542	28.98444	25.85809	28.16338	29.25032	30.84515	30.17563	29.16799	29.77893	32.29275	26.92736	30.09551	30.05381	30.35315	31.47994	29.78496	27.78496
hsa-miR-605	38.89225	38.70512	38.32448	37.98623	36.87572	34.23734	34.17001	32.3357	38.80757	38.11424	36.61058	36.85348	36.08793	38.75356	37.08973	38.82518	36.1274	35.16719	37.29751	37.62966
hsa-miR-766-3p	30.69187	32.88527	29.49879	30.04135	30.12567	29.89146	29.9058	29.03381	31.81182	31.29208	30.40159	30.85566	33.94442	28.95795	31.05029	30.53629	31.54236	32.47547	31.93192	28.87702
hsa-miR-19a-3p	23.4563	22.99221	22.72103	23.34391	21.25695	22.69854	23.66016	22.38645	22.38587	24.07519	23.62305	23.16023	22.65393	23.99387	23.48868	22.26454	22.78923	23.12068	23.21873	21.77285
hsa-miR-584-5p	32.79792	34.59336	32.19546	32.4045	33.72847	30.19091	32.71464	32.9657	33.51759	34.21453	33.27483	33.9204	34.42934	32.15987	34.59285	33.6272	35.33808	33.69693	33.16641	31.99856
hsa-miR-144-3p	26.69103	25.947	25.61288	26.22725	24.15369	27.59957	26.74652	26.32333	25.81713	26.99054	26.81098	25.96434	25.93203	27.29754	27.66445	25.47022	26.14856	26.47072	26.19278	24.7817
hsa-miR-92a-3p	22.52407	22.46799	22.06299	22.27347	20.78026	22.35568	23.22431	21.53899	21.73056	23.15031	22.5224	21.67895	22.20973	23.16956	22.69553	21.71139	22.46906	22.61225	22.38742	21.38729
hsa-miR-126-3p	25.55144	26.61297	24.1063	25.10461	23.72582	21.56704	24.56669	23.62752	24.78778	25.95621	24.77132	25.35007	26.16188	24.13579	24.80885	25.40429	26.07971	25.45447	25.6893	23.6741
hsa-miR-363-3p	28.56565	27.33586	27.26963	27.90775	25.09547	28.43548	28.62302	27.64384	27.18851	28.75017	27.54857	27.94684	27.62754	28.39699	29.18393	27.60034	28.27741	27.83662	28.50162	26.44453
hsa-miR-148a-3p	29.12604	29.94817	28.70185	29.29599	25.24286	27.40551	28.23301	28.72833	29.0815	29.67859	28.44513	27.77618	28.80755	27.72924	29.88059	28.38501	29.51613	29.35132	29.26181	27.9475
hsa-miR-374a-5p	32.40572	32.36877	31.07847	31.19238	29.85797	29.19226	31.73932	33.34733	32.35363	32.97143	31.38966	31.58953	34.5748	31.14826	33.0492	33.37634	33.82884	33.61235	31.79604	30.59642
hsa-miR-10b-5p	33.67074	33.98075	31.67362	32.04171	30.7248	33.58717	32.11369	30.90057	33.38485	32.66679	31.65718	31.30852	31.33387	32.53956	33.38741	31.4356	32.58463	31.08337	32.03518	32.70745
hsa-miR-190a	36.72818	35.44731	34.60999	35.72492	34.44106	36.20168	38.00856	38.32327	37.00179	36.15786	35.56296	39.9922	36.96162	36.58842	37.8623	35.64421	37.90305	38.90798	37.13423	36.02359
hsa-miR-195-5p	36.76088	37.41334	35.18873	35.27494	33.91811	33.00945	34.59364	34.93774	36.36157	36.00436	34.81803	36.10756	33.75375	34.36822	37.39847	35.40641	36.0197	34.40466	35.86294	33.09562
hsa-miR-29a-5p	34.49987	35.70321	32.62996	35.66669	31.3966	31.3523	33.67048	31.63377	33.19236	35.6004	33.62799	34.79367	34.28976	33.2996	33.22906	34.80042	38.57678	37.89062	37.7499	37.23841
hsa-miR-125b-5p	29.24719	30.91387	28.41159	29.92798	24.82638	28.13419	29.30256	27.91069	29.80998	29.88473	28.15728	27.19051	29.76214	29.76095	27.19072	29.53757	30.33122	30.34946	28.97484	28.53735
hsa-miR-18a-3p	32.28524	31.58624	31.78024	31.89217	30.31439	31.42086	33.18858	31.88136	32.14621	33.06258	32.00536	32.00099	32.72459	32.47067	32.8706	31.53223	32.61909	33.26028	31.60923	31.05726
hsa-miR-192-5p	29.07099	27.54689	27.89643	28.37286	23.74936	28.63328	28.94552	27.89466	27.20621	29.42422	27.34306	26.18886	28.63762	28.85185	28.05868	27.59172	28.44694	28.99431	28.66389	26.8717
hsa-miR-151a-3p	31.51415	32.30343	30.65316	31.9832	30.03455	27.4612	30.95442	31.12581	32.31164	32.39593	30.55849	32.06683	32.0797	30.09848	32.8249	31.15724	32.20752	32.96207	31.79728	30.82891
hsa-miR-18b-5p	28.99933	27.94695	27.48807	28.08904	26.35813	26.48362	28.52447	27.22943	27.34803	29.13809	28.40923	27.97903	29.01157	28.29912	28.93529	28.25493	29.48258	29.40135	28.55834	26.62752
hsa-miR-28-3p	25.39149	32.65604	29.45499	30.75947	28.58719	27.13393	29.81975	28.47832	31.05596	31.50272	29.5291	30.47097	31.39117	28.99073	30.75623	29.88137	32.48067	31.30003	30.68423	29.13814
hsa-miR-335-5p	32.17131	33.31935	30.9563	31.99035	30.49982	28.57853	31.68173	29.35351	31.32729	32.61351	32.37259	32.34742	32.77194	31.03586	31.78023	31.68464	31.80368	32.62725	32.28526	30.70428
hsa-miR-324-3p	28.5075	27.65919	27.27883	27.91063	25.89974	26.96228	28.57415	27.8928	27.26611	28.83177	27.76575	27.52392	28.42872	28.24235	28.56025	27.25133	28.24845	28.6657	28.26685	26.59406
hsa-miR-204-5p	32.6358	34.69013	32.43379	31.41585	29.61652	32.08724	32.84146	30.96939	34.26277	34.95685	33.90165	31.81868	34.49849	33.61979	35.21167	32.12917	33.18265	34.07457	33.65804	33.79034
hsa-miR-182-5p	37.10955	34.85246	36.65845	37.84937	33.86834	35.53553	39.14747	37.98629	37.98236	36.9383	37.04663	36.20702	38.41929	36.38702	36.88975	38.07404	36.89752	36.86781	36.41492	35.64703
hsa-let-7g-5p	26.46112	25.72285	25.48306	25.61098	23.52012	23.27145	25.88853	24.8771	24.92423	26.6523	25.08185	25.44943	27.26788	24.88479	26.01689	25.97483	27.30828	26.98093	26.3918	26.56339
hsa-miR-15b-5p	27.54903	26.97654	26.12947	26.97556	24.46608	24.49806	27.23634	26.48847	26.40215	27.87269	26.73571	27.12242	27.42981	25.85077	27.16329	26.56068	27.54486	27.3851	26.55589	25.73171
hsa-miR-22-3p	26.27411	25.47344	24.95457	26.14259	23.49456	24.17081	25.70176	24.98878	25.41715	26.65558	26.2033	25.44125	26.40162	25.58852	27.39152	24.8139	25.51819	25.85736	24.90096	23.78973
hsa-miR-106b-3p	32.81747	32.63624	31.18234	31.35515	30.10227	28.63553	31.69542	31.26469	31.61597	33.11651	31.51329	32.23705	32.53256	30.10619	32.31112	32.73843	32.62348	33.9879	31.5128	30.99951
hsa-miR-199a-3p	28.62543	31.2943	27.35934	28.50399	27.16084	24.56451	27.31032	27.04197	28.37663	29.06122	27.75777	28.90052	30.13633	26.03986	28.54507	28.54625	29.527	29.5624	28.6566	27.12385
hsa-miR-29c-3p	28.07731	27.83083	27.13386	28.44283	25.37944	26.94735	27.86223	26.73084	26.99192	29.3093	27.72294	27.45336	27.93699	27.47156	28.3465	26.8371	27.41397	28.34147	27.19159	26.35056
hsa-miR-19b-3p	22.80819	22.26149	21.96556	22.62489	20.70661	22.05826	22.90886	22.18024	22.0219	23.46206	22.70167	22.34631	22.06381	23.1515	23.3283	21.66688	22.20088	22.57058	22.45527	21.048
hsa-miR-95	35.35854	36.1527	36.6823	38.38045	33.2107	35.79063	34.90127	35.84692	36.81309	35.6254	36.1368	35.17346	36.75823	37.03688	37.23497	35.45067	37.7169	35.09236	34.45426	35.05575
hsa-miR-29a-3p	29.70453	29.75754	27.37718	28.99457	25.45903	26.8369	28.18458	27.70327	28.52023	29.59363	27.75886	27.77	29.27981	26.70421	28.96121	28.35319	29.33983	29.43939	28.56105	27.39969
hsa-miR-21-5p	25.14946	25.01872	23.84129	24.94272	23.11337	22.66476	23.74803	23.78481	25.19405	25.52586	24.7081	24.43927	24.95989	23.85697	25.72037	24.31495	25.75683	25.00069	25.0331	23.75778
hsa-miR-150-5p	26.68782	26.66686	24.1158	24.84476	23.71416	25.22556	26.58527	24.3579	23.57379	26.28103	22.86076	23.9268	23.27749	23.41396	24.21265	25.07351	25.86813	26.33533	25.59059	24.73113
hsa-miR-30e-3p	33.98795	34.48735	31.56219	33.11497	29.97708	29.58775	32.30341	32.67778	33.89531	34.38569	32.33401	31.9388	35.73007	30.9821	33.88789	33.25337	34.48325	33.59615	32.93573	31.38476
hsa-miR-30b-5p	27.96956	27.70412	26.51385	26.71822	24.37031	24.55574	26.99615	26.62218	27.21026	28.1143	26.5017	26.73009	28.80591	25.81084	27.85484	27.54214	28.84336	28.16653	27.47687	25.52254
hsa-miR-543	33.3403	38.75872	35.35503	33.76499	34.31726	29.09432	33.78001	32.57128	35.80063	37.94935	33.20444	38.4225	34.60691	31.31915	31.77762	34.07417	34.77639	34.38651	33.29401	32.80764
hsa-miR-24-3p	25.31418	26.14055	23.99253	25.09914	22.29473	21.4738	23.97835	23.0359	23.95369	25.2284	24.25223	24.8208	25.77845	23.43045	24.0378	24.548	25.13255	25.36255	24.97988	23.30529
hsa-miR-29b-2-5p	33.70686	32.81276	32.68963	33.68156	31.17325	31.31618	33.12174	31.14022	33.00141	33.61847	32.35545	32.50351	33.15458	33.26297	33.16234	31.89248	33.61815	34.13213	33.03684	31.70088
hsa-miR-495-3p	34.7331	35.84284	34.12085	32.59601	33.4417	28.66639	32.39975	32.26211	35.36662	34.84154	31.52793	33.24242	31.32449	31.03782	32.51954	33.92587	35.08429	33.27911	33.34108	32.02823
UniSp3 IPC	20.74053	20.88739	20.8411	20.73717	20.82012	20.95567	21.05521	21.24502	21.54098	20.82124	20.91285	20.82769	20.81447	20.79199	21.49244	20.83724	20.87916	20.95243	20.74931	21.03314

### SUPPLEMENTARY PROCEDURES

#### miRNA qRT-PCR array

A total of 192 mature human miRNAs were detected in the plasma samples. Specifically, the reaction consisted of 4 μl diluted cDNA template, 5 μl SYBR Green master mix, and 1 μl PCR primer mix (Exiqon A/S). The reaction was denatured at 95°C for 8 min, followed by 36 cycles at 95°C for 10s, and at 60°C for 60s. Melt curve analysis was performed at the end of the PCR.

#### RNA extraction and assay of real-time PCR

TRIZol reagent (Thermo Fisher Scientific) was used to isolate total RNA from HEK-293T cells, followed by reverse-transcription with a PrimeScript^TM^ RT Reagent Kit, according to the manufacturer's instructions and as we previously described with several modifications (1-5). mRNA expression level of DRD1 and GAPDH was determined by RT-PCR with Power SYBR Green PCR Master Mix (Thermo Fisher Scientific) in an ABI Prism 7900 (Applied Biosystems), following the manufacturer's protocol. Data were normalized against the mean CT value of GAPDH, and the relative mRNA expression level was determined by the 2^−ΔΔCT^ method, as we previously described (5-11).

#### Validation of miRNA expression by qRT-PCR analysis

miR-197-5p, miR-130a-5p, miR-27a-5p, and miR-195-5p were chosen for verification with the qPCR array data by RT-qPCR. The above miRNAs were selected based on expression levels and biological significance. The miScript Reverse Transcriptase Kit (Qiagen, Germany) was used to perform cDNA synthesis, following the manufacturer’s instructions. In brief, an RT reaction consisting of 1 μg RNA, 2 μl of Nucleics Mix, 2 μl of miScript Reverse Transcriptase Mix, and 4 μl of miScript HiSpec Buffer was created. The reaction was incubated at 37°C for 60 min, at 95°C for 5 min, and then held at 4°C. A miScript SYBR^®^-Green PCR kit (Qiagen GmbH) was adopted to perform qPCR. The reaction system contained 10 μl RT-qPCR mix, 0.5 μl upstream primer (miRNA-197-FW: 5'-TGCGGCGGGTAGAGAGGGCAGTGG-3'; miRNA-130a-FW: 5'-TGCGGTTCACATTGTGCTACTGTCTGC-3'; miRNA-27a-FW: 5'-TGCGGAGGGCTTAGCTGCTTGTGAGC-3'; miRNA-195-FW: 5'-TGCGGTAGCAGCACAGAAATATTGGC-3'), 0.5 μl downstream primer (common primer supplied by the kit), 7 μl ddH_2_O, and 2 μl template of cDNA. The steps of thermal cycle reaction are as follows: initial denaturation for 10 min at 95°C, 38 cycles for 1 min at 95°C, and 30s at 60°C. Calculation by the 2^-ΔΔCt^ method (20) was performed to evaluate the expression level of miR-199a-3p normalized to U6 (forward primer, 5'-GCTCGCTTCGGCAGCACA-3' and the reverse primer, 5'-AACGCTTCACGAATTTGCGTG-3').

#### Cell culture and transfection

HEK-293T cells were cultured in DMEM (Invitrogen; Thermo Fisher Scientific, Inc.) supplemented with 10% FBS, 100 units/ml Invitrogen penicillin-streptomycin (Thermo Fisher Scientific, Inc.) in a humidified incubator with 5% CO_2_ at 37°C as we previoSSusly described (12,13). miRNA was transiently transfected with Lipofectamine 2000 reagent, according to the manufacturer's instructions (Invitrogen). All experiments were done in triplicate.

#### Luciferase reporter assay

The miR-195-5p mimic and miR-195-5p inhibitor, and their negative control mimics were purchased from GenePharma (Shanghai, China). Chemically-modified miRNA inhibitors were optimized to specifically inhibit miR-195-5p expression. miRNA mimics and inhibitors were transfected into HEK-239T cells, as described above. At 24h, the transfected HEK-293T cells were used for the luciferase report assay and target mRNA expression detection as we previously reported (12,13).
